# Epidemiology, disease burden and costs of Duchenne muscular dystrophy in Germany: an observational, retrospective health claims data analysis

**DOI:** 10.1186/s13023-025-03906-x

**Published:** 2025-08-13

**Authors:** Joanna Diesing, Janbernd Kirschner, Astrid Pechmann, Jörg König, Leonie Kunk, Tarcyane Barata Garcia, Carolina Schwedhelm, Carsta Militzer-Horstmann, Ivonne Hänsel, Agnes Kisser

**Affiliations:** 1 WIG2 GmbH, Scientific Institute for Health Economics and Health System Research, Markt 8, 04109 Leipzig, Germany; 2 Department of Neuropediatrics and Muscle Disorders, Faculty of Medicine, Medical Center – University of Freiburg, Breisacher Str. 62, 79106 Freiburg, Germany; 3 Pfizer Pharma GmbH, Friedrichstraße 110, 10117 Berlin, Germany

**Keywords:** Duchenne muscular dystrophy, Prevalence, Germany, Real-world data, Algorithm, Disease stages, Costs, Healthcare resource utilization, Epidemiology

## Abstract

**Background:**

Duchenne muscular dystrophy (DMD) is a rare genetic disorder that primarily affects males. Beginning in childhood, patients experience ambulatory loss, heart failure and need ventilation. Disease management has improved, however, DMD remains debilitating, and has no cure. The rarity of the disease makes research difficult, and German prevalence data are lacking. Cost and resource utilization estimations are based on small sample sizes or self-reported data, limiting generalizability and adds the potential for recall bias. With a retrospective study on a healthcare claims database, we adapted algorithms to identify DMD patients and categorized them by disease stages 1–4 (early ambulatory, late ambulatory, early non-ambulatory, late non-ambulatory) with increasing disease progression. We analyzed annual prevalence, burden of disease, healthcare resource utilization and direct medical care costs, by time under observation (patient year).

**Results:**

From 2016 to 2021, we identified 134 patients for which we could determine a disease stage and determined an extrapolated prevalence of DMD in Germany between 14.85 (95%CI 12.17, 17.95) and 18.91 (95%CI 15.70, 22.61) per 100,000 males under 40 years of age. Most patients we identified met DMD stage 4 group criteria (4*7*.01%), followed by stage 3 (3*7*.31%), stage 2 (33.58%) and only 4.48% in stage 1. The average age increased with progressing disease, from 4.27 years in stage 1, to 11.43, 18.86 and 23.21 in stage groups 2, 3 and 4, respectively. In the stage 2 group, diagnosis codes reflecting mobility support and orthopedic surgical interventions (15.56% of the group) were documented. In the stage 3 group, decubitus prevention was documented, increasing to around half of patients in the stage 4 group. Total direct mean healthcare costs per patient year increased substantially with disease severity group, from €2,180.73 (SD 16,258.90) in stage 1; €13,599.83 (SD 33,756.07) in stage 2; €14,472.08 (SD 27,245.78) in stage 3 and finally €41,888.70 (SD 117,718.13) in stage 4. Especially in stage groups 3 and 4, medical aids accounted for about half of total costs.

**Conclusions:**

We present an algorithm on which further research can be based, and provide a current picture of epidemiology, burden of disease and healthcare utilization and direct costs of DMD in Germany.

**Supplementary Information:**

The online version contains supplementary material available at 10.1186/s13023-025-03906-x.

## Background

Duchenne muscular dystrophy (DMD) is a rare, severe, and life-threatening x-linked genetic disorder that primarily affects boys [1]. The characteristic progressive muscle tissue loss begins with muscle weakness in early childhood; the first symptoms often manifest between two to three years of age [2] and mean age at diagnosis is 3.8 years (± 2.4) [3]. The course of DMD is characterized by irreversible progressive deterioration of motor function and other organ system function leading to heart failure, impaired breathing, and need for ventilation [4] and ultimately premature death [1, 5]. By the early teen years, DMD patients are confined to a wheelchair [4–6] and rarely live beyond the age of 40 [7, 8]. Progression of the disease can be roughly categorized in four disease stages: in the early ambulatory stage, patients may exhibit early signs of proximal muscular weakness, but the risk of respiratory, cardiac, or gastrointestinal symptoms is low [9]. Treatment and risk assessment can include implementing drug therapies, vaccination protection, and echocardiography [10]. In the late ambulatory phase, progressive loss of muscle function becomes increasingly evident, often necessitating assistive devices. Surgical intervention may be considered to improve function as needed. Cardiac dysfunction may be managed with angiotensin converting enzyme (ACE) inhibitors or angiotensin receptor blockers (ARBs) from the age of 10 years onwards [10]. The risk of respiratory issues remains low at this stage [9]. The mean age of entering non-ambulation is 11 years [6] and from this point on or even earlier, most patients are dependent on a wheelchair [11]. In the early non-ambulatory phase, orthopedic management is of focus, with surgical interventions for contractures or scoliosis [10]. Finally in late non-ambulatory phase, upper limb function becomes significantly impaired and patients are confined to a bed [8], and there is a high risk of respiratory complications and worsening cardiomyopathy [9]. Respiratory support measures including cough-assist devices, nocturnal ventilation, and potentially daytime ventilation may be needed [10].

Available therapeutic options aim to maintain function and prevent fixed deformities, contractures and pressure and pain, however there is no cure [7]. Initial drug therapies are glucocorticoids (deflazacort and prednisone) [5–7] and can include ataluren (Translarna®). However, the latter was only on a conditional marketing authorization for a selection of DMD patients with nonsense mutations, which the European Medicines Agency decided not to renew in March 2025 [12].

Estimating epidemiological and cost data for DMD is challenging. Health claims data are a valuable resource for large scale analyses but present difficulties in identification of a DMD study population. A 2020 systematic review estimated the global pooled prevalence to be 7.1 (95% CI 5.0, 10.1) per 100,000 males [13] with diagnosed prevalence in Europe estimated at 6.5 (95% CI 5.0, 8.2) per 100,000 males, however specific prevalence data for Germany are lacking. Furthermore, German cost and HCRU (healthcare resource utilization) estimations are necessary for stakeholders; 2013 mean direct costs were estimated at €19,346 (ranging from €4,420 to €68,968, depending on the disease stage) [8]. However, these were based on small sample sizes or self-reported data from patients and/or families, introducing the possibility of recall bias [8].

The goal of our study was to evaluate the epidemiology, HCRU and costs of the DMD population, by disease stage, in Germany. Our work employs an approach tailored to German health claims data, to define and analyze DMD patients within a healthcare claims database, addressing the gap in German DMD research through innovative methods.

## Methods

This real-world descriptive observational study in a large claims database in Germany covered the years 2016–2021. The study design included both cross-sectional and longitudinal elements to identify patients with DMD, then analyze burden of disease, HCRU and direct healthcare costs by disease stage.

### WIG2 database description

The WIG2 research database is an anonymized healthcare claims database in Germany with longitudinal inpatient and outpatient care data from about 4.5 million patients insured by the German statutory health insurance (SHI) funds, which represents approximately 90% of the German population [14]. The dataset is representative of the German SHI population with regard to sex, age, and morbidity [15]. Available data includes diagnosis codes (ICD-10) for both outpatient and inpatient visits [16], as well as Anatomical Therapeutic Chemical codes (ATC) for medications [17], German Uniform Assessment Standard (Einheitlicher Bewertungsmaßstab, EBM) codes for medical aids [18], and German Procedure Classification of operations and procedures (Operationen- und Prozedurenschlüssel, OPS) codes [19]. Direct costs are reported as the amounts paid by the SHI fund. No primary patient data was collected for the conduct of this research, and ethics approval of these secondary data is not required, as stipulated by the 10th chapter of book V of social code in Germany.

### Patient selection

For DMD population selection, we adapted previously developed claims-based algorithms [20–22] combined with expert opinion, to identify DMD patients and disease stages within health claims data, leveraging distinctive characteristics of DMD to differentiate it from other muscular dystrophies in the German healthcare context. The ICD-10 code G71.0 used for muscular dystrophies in Germany includes several conditions beyond DMD, such as Becker muscular dystrophy, limb-girdle muscular dystrophy, scapuloperoneal muscular dystrophy, oculopharyngeal muscular dystrophy and facioscapulohumeral muscular dystrophy [14].

**Fig. 1:**
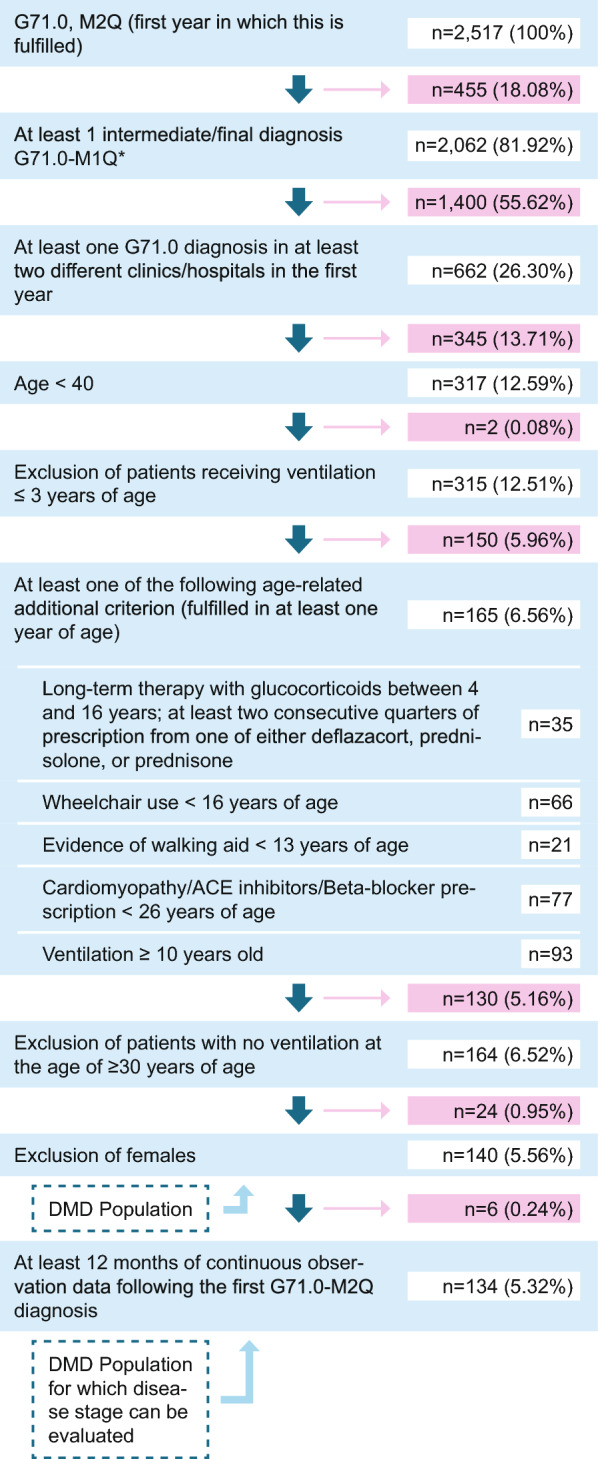
Flow diagram of inclusion criteria/algorithm to identify DMD patients (n, %) at each step Abbreviations: n: number of patients

Our algorithm is displayed in Fig. 1 and described in more detail in Supplementary Table 1. In addition to fulfilling M2Q (minimum 2 quarters) criterion for code G71.0 (either two verified outpatient/secondary inpatient diagnoses in different quarters or one main inpatient diagnosis), patients were required to have one final diagnosis (step 2) during the longitudinal follow-up period, except for patients included in 2021, for which no follow-up years/diagnoses were possible. Also, patients with gap years in between the final diagnosis were included. Age-related criteria were employed in the patient identification algorithm to differentiate patients with DMD from other muscular dystrophies. For example, symptom onset and loss of ambulation occur at a later stage in Becker muscular dystrophy, and life expectancy is greater than in DMD [23]. Considering a median life expectancy of DMD patients of 28.1 years (95% CI 25.1, 30.3) [7], we included patients up to age 40 to capture the full range of DMD stages. Older patients were excluded due to the likelihood of a different disease rather than DMD, given the typical progression of DMD [7, 8]. We then excluded patients with documentation of ventilation at three years of age or younger, since this would be very atypical of DMD [24]. To be included in the DMD study population, patients had to fulfil at least one of the following additional age-related criteria (fulfilled at least once in the observation period): long-term therapy with glucocorticoids between 4 and 16 years, determined by at least two consecutive quarters of prescription from one of either deflazacort, prednisolone, or prednisone [25, 26]; evidence of wheelchair use by the age of 16 years; evidence of walking aid by the age of 13 years [4]; diagnosis or medical treatment for cardiomyopathy (ACE inhibitors/Beta-blocker prescription) by the age of 26 years; and finally any evidence of ventilation support from the age of 10 years [24]. A complete list of codes used to determine inclusion and exclusion criteria is available in the supplement (Supplementary Table 2).

We then excluded remaining patients who were not receiving ventilation over the age of 30, and any females remaining, for our final DMD population.

### Classification by disease stage

For DMD disease stage approximation, we adapted a claims-based algorithm developed by Iff et al. (2022) [27] to the German context, using a targeted literature review [28, 29] and expert input. We applied criteria only to patients in our DMD population with at least 12 months of data, to ensure disease stage assessment was based on sufficient data.

We determined disease stage based on a series of criteria (Table 1), categorizing the stages into early ambulatory, late ambulatory, early non-ambulatory, and late non-ambulatory. Patients were allocated to the highest stage, for which they met the criteria, and followed longitudinally across the time frame 2017–2021, until they met the criteria for a higher stage. During this time, patient data (age, clinical characteristics, HCRU, and cost) was recorded. When further stage criteria were met, the same patient data was recorded under that stage and, until the patient was no longer in the database, was deceased, turned 40 years of age, or moved onto a further disease stage. To ensure there was enough data to reliably allocate a patient to a disease stage, a minimum of 12 months of data following M2Q diagnosis criteria was required. Some patients could not be allocated to a disease stage, either because of insufficient (< 12 months) follow-up data, or because the patient did not fulfill any of the disease stage criteria during 12 months of data collection. A full list of codes used for the disease stage allocation criteria can be found in the supplement (Supplementary Table 3).

**Table 1:** DMD stage identification criteria

Step	Details
*Stage 1: Early Ambulatory*	
1	Lack of criteria from other disease stages, and < 8 years of age
*Stage 2: Late ambulatory: any one of the following criteria (except for #2; only in combination with #1*)	
1	Long-term therapy with glucocorticoids (at least two consecutive quarters of prescription from one of either deflazacort, prednisolone, or prednisone)
2	ACE/ARB, Beta-Blocker prescription, or cardiomyopathy diagnosis, in combination with long-term therapy with glucocorticoids (#1)
3	Scooter, or wheelchair pre scription (manual) or evidence of walking aid
4	Orthotic or prosthetic therapy (footplate, ankle motion, innerboot) and 9–13 years of age
*Stage 3: Early non-ambulatory: any one of the following criteria*	
1	Powered wheelchair
2	Mobility assistance
3	Pulmonary management: nasal-, positive airway and breathing devices
4	Scoliosis therapy
5	Hospital bed or mattress
6	ACE/ARB, Beta-Blocker prescription or cardiomyopathy diagnosis
*Stage 4: Late non-ambulatory: any one of the following criteria*	
1	Defibrillator, ventricular support, heart transplant
2	Tracheostomy and related products
3	Ventilation support and related devices
4	Enteral formular, nutrition infusion pump and ≥ 10 years of age
5	PEG/PEJ tube

ACE/ARB: Angiotensin-converting enzyme inhibitor/angiotensin receptor blocker; PEG/PEJ tube: Percutaneous endoscopic gastrostomy/percutaneous endoscopic jejunostomy

### Burden of disease, HCRU and direct healthcare costs

To assess DMD patients’ burden of disease, we analyzed the 20 most frequently documented concomitant diagnoses, medications, medical aids and operation/procedure codes in the DMD population by disease stage.

Diagnoses in the research database can be traced back to the sector in which they were documented (such as outpatient or inpatient, pharmacy outpatient), and HCRU and costs are reported accordingly. We also identified cardiac and respiratory management costs based on pre-selected codes determined in cooperation with medical experts and based on findings from the literature. The pre-defined codes used can be found in Supplementary Table 3 and include ICD-10 diagnosis, OPS procedure and SHI medical aid codes for respiratory management, and ICD-10 diagnosis and ATC medication codes for cardiac management.

### Statistical methods

In the cross-sectional analysis of the study, DMD prevalence in the WIG2 database was calculated as the fraction of patients that met our DMD population criteria, of all males in the WIG2 database between the ages of 0 and 39 (by 100,000 males in the same age and sex strata). The extrapolation to the German population was done by calculating the weighted averages of the stratum-specific rates in the WIG2 study population, using the corresponding number of each in the standard population as weights. These direct adjustment weights were calculated by dividing the number of patients in each stratum-specific group in the German population, by the same number of patients in the WIG2 research database, with 95% CI and standard deviation (SD), the former estimated according to Fay & Feuer [30].

In the longitudinal analyses (burden of disease, HCRU and costs), patients were followed from the time they met criteria for the DMD population, and a disease stage was assessed (after 12 months of follow-up time) to the time they left the cohort or met criteria for a subsequent disease stage. The age data (mean, median, first and third quartiles, min/max, and SD) was reported for the whole population, and by disease stage.

To estimate burden of disease in DMD patients, documented codes were evaluated by disease stage; in a longitudinal analysis, the 20 most frequently documented codes were reported by disease stage, specifically 3-digit ICD-10-GM codes, 5-digit ATC codes, 4-digit EBM codes, and 3-digit OPS codes. When patient numbers were < 5, a number of patients and proportion could not be reported, due to regulations around data protection, and for simplicity we did not report these codes, unless the code was documented in ≥ 5 patients in another disease stage, we marked that it was among the reported codes but with < 5 patients.

HCRU and costs were then reported by disease stage in the study population, including number of inpatient days, hospital admissions, outpatient visits, emergency department visits, number of outpatient pharmacy prescriptions, number of physio- and occupational therapy cases, and the number of medical aid prescriptions. Additionally, costs were reported for cardiac and respiratory management and all costs were also reported by age group (0–3 years, 4–7 years, 8–12 years, 13–17 years, and ≥ 18 years). The mean number of resources were presented as per patient year (PPY), a measure that accounts for the different amount of time spent by patients in the disease stages and in the study, with the SD. This data was reported both as mean among all patients, and as the mean among only patients with at least one documentation of the respective HCRU.

## Results

### Patient characteristics

Of the 2517 patients in the WIG2 database with documented G71.0 diagnosis, our patient identification algorithm resulted in a DMD population of 140 males. Of these, 134 had at least 12 months of follow-up data, for determining disease stage and we were able to assign between 99 and 119 DMD patients to a disease stage, depending on observation year (see Supplementary Table 4 for data on patients included by year).

**Fig. 2:**
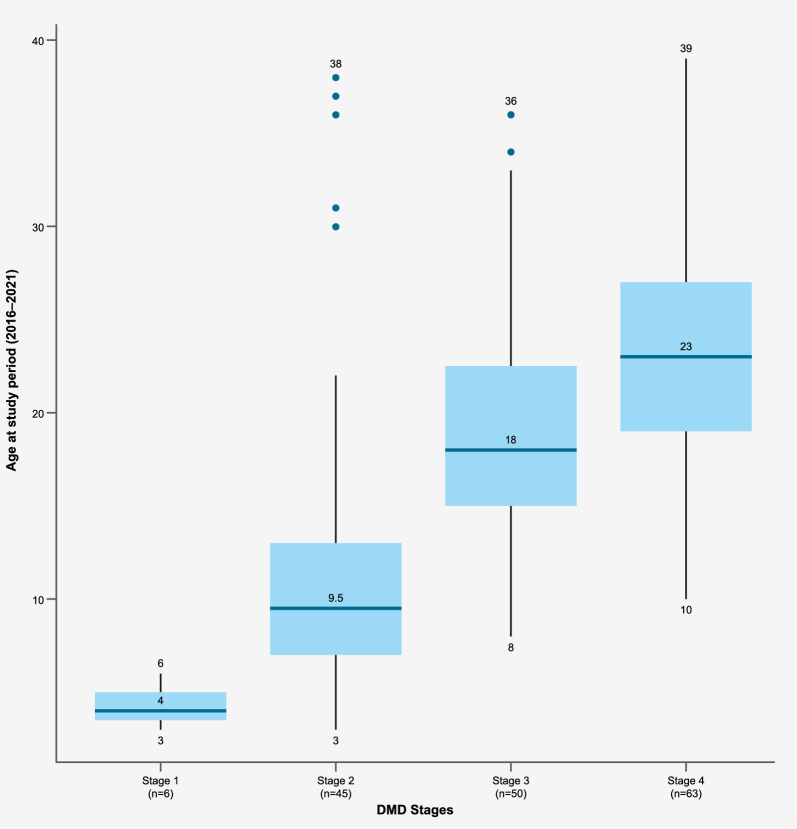
Box plot of age by DMD disease stage criteria, longitudinally collected across 2017–2021 (n = 134) Abbreviations: DMD: Duchenne muscular dystrophy

Across the timeframe 2017–2021, of the 134 patients allocated to a disease stage, some of these patients were assigned to more than one disease stage (consecutively) throughout these five years. DMD patients were older with increasing disease stage (Fig. 2), with 4 (4–5), 10 (7–13), 18 (15–23), and 23 (19–27) years of age (median and interquartile range, IQR) in stages 1, 2, 3 and 4, respectively (Table 2). A similar distribution was observed when considering individual study years. There was some overlapping of age groups among disease stages; maximum age reached 38, 36 and 39 years in disease stages 2, 3 and 4, respectively, although the third quartile values were consistently older with each stage.

**Table 2:** Patients by DMD disease stage classification, year (cross-sectional analysis) and mean age (standard deviation)

Year	2017–2021		2017		2018		2019		2020		2021	
All patients in this cohort	134		116		118		118		125		126	
Patients by stage N* (%)												
Stage 1	6 (4.48%)		6 (5.17%)		< 5		< 5		< 5		0 (0.00%)	
Stage 2	45 (33.58%)		21 (18.10%)		29 (24.58%)		28 (23.73%)		27 (21.60%)		27 (21.43%)	
Stage 3	50 (37.31%)		29 (25.00%)		27 (22.88%)		29 (24.58%)		30 (24.00%)		36 (28.57%)	
Stage 4	63 (47.01%)		43 (37.07%)		49 (41.53%)		50 (42.37%)		53 (42.40%)		56 (44.44%)	
*Age by DMD stage*												
	Mean (SD)	Median (IQR)	Mean (SD)	Median (IQR)	Mean (SD)	Median (IQR)	Mean (SD)	Median (IQR)	Mean (SD)	Median (IQR)	Mean (SD)	Median (IQR)
Stage 1	4.27 (0.98)	4 (4–5)	3.83 (0.94)	4 (3–5)	- ( -)	- (–)	- ( -)	- (–)	- ( -)	- (–)	0.00 (0.00)	- (–)
Stage 2	11.43 (6.03)	10 (7–13)	12.36 (6.48)	9 (7–15)	11.54 (7.18)	9 (7–13)	10.95 (6.23)	9(7–11)	11.45 (6.29)	10 (8–12)	11.07 (3.42)	11(8–13)
Stage 3	18.86 (5.84)	18 (15–23)	18.52 (5.40)	18(15–24)	18.52 (5.46)	18(15–21)	18.52 (6.06)	18(14–21)	18.67 (5.60)	18(14–22)	19.83 (6.44)	19(15–23)
Stage 4	23.21 (6.11)	23 (19–27)	21.98 (6.31)	21 (18–26)	22.43 (6.10)	22 (18–26)	22.82 (5.67)	23 (18–26)	23.72 (5.91)	23 (20–27)	24.71 (6.27)	24 (21–28)

DMD: Duchenne muscular dystrophy; IQR: Interquartile range; SD: standard deviation

^*^N reports the number of patients that were assigned to the stage throughout the timeframe indicated; note that some patients could have been assigned to more than one stage in the timeframe (consecutively), and not all patients in the cohort could be assigned a disease stage

When there were < 5 patients in the selected subgroup, no data could be reported for data protection reasons

### Prevalence

**Fig. 3:**
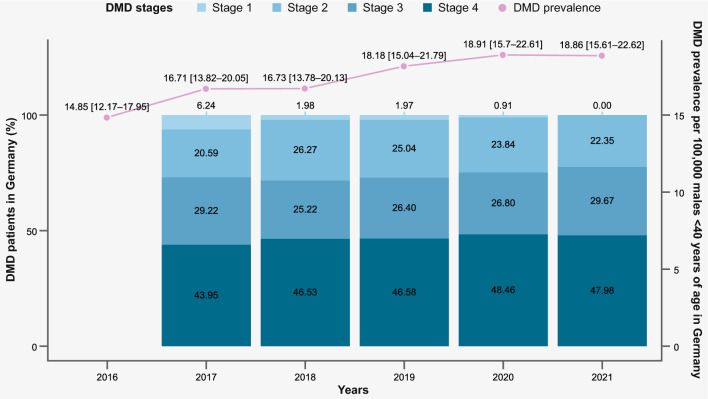
DMD prevalence and proportion (%) of patients by disease stage allocation/year (2016–2021) in Germany *Note: per 100,000 male patients < 40 years of age, with 95% confidence intervals. In 2016, there was not yet enough observation time to determine patients’ DMD disease stage, therefore information is only available from 2017 onwards

We found a prevalence range of 14.85 DMD patients in Germany (95% CI 12.14, 17.95, in 2016) to 18.91 (95% CI 15.70, 22.61, in 2020) patients per 100,000 German males < 40 years old (Fig. 3).

Prevalence of patients in stages 2 and 3 was similar; in any given year there were nearly twice as many patients in disease stage 4, compared to stages 2 or 3. There were very few patients identified in stage 1, with at most 2% of DMD patients, or even none in 2021, that met these criteria. The proportion of DMD patients who could not be assigned to a disease stage was up to 11% (in 2020), depending on the observation year, with the lower range consisting of n < 5 patients.

### Burden of disease and HCRU

In the stage 1 group, we saw diagnosis codes indicating motor function or general developmental disorders, and speech articulation disorders. Many codes included need for immunization or acute respiratory infections. Few patients met stage 1 criteria; therefore, we had little data available and thus few documented codes. There were < 5 patients with medications documented, including glucocorticoids and some pain medications, with an average of 6.82 (SD 13.93) prescriptions PPY. There was little use of medical aids in this group, only prescriptions for foot support (orthoses, insoles or shoes), and no operations, procedures, hospital admissions or inpatient days were documented. This group had a mean of 8.91 outpatient visits PPY (SD 11.88), which was a similar amount as other stage groups, and most (83.33%) had at least one physio- or occupational therapy prescription with 2.36 cases on average, PPY (SD 4.47).

In our stage 2 group, we observed more DMD-indicative codes, such as muscle disorders, gait and mobility abnormalities, and developmental and motor disorder codes were documented in nearly half of the group. There were also codes indicating pain medications and dependence on enabling machines and of limb deformities, and a higher use of glucocorticoids (68.89%) than in stage 3 group (24%) was observed. A fifth of the stage 2 group were taking ACE-inhibitors, contributing to the use of cardiac healthcare by about a third of this group.

As per our disease stage criteria, we saw use of mobility support beginning at stage 2, with around 60% of patients having disabled vehicle aids, with a mean of 3.63 medical aid prescriptions PPY (SD 7.64). Orthopedic surgical interventions were documented in 15.56% of this group, with a similar proportion prescribed pneumological function tests (17.78%). This group used outpatient healthcare the most of all others, with a mean of 12.69 visits PPY (SD 20.59).

Our stage 3 group had codes for scoliosis (38.00%), cardiomyopathy (28.00%), and heart failure (20.00%) documented. Medical aid use increased further in the stage 3 group, with a mean 6.16 prescriptions (SD 10.92) with 16.00% of the stage 3 group prescribed decubitus prevention medical aids, and over half (up to 68.00%) with disabled vehicle aids. Use of incontinence and toilet aids, respiratory therapy devices and arm orthoses were documented more frequently starting in this disease stage group. We also found that 18.00% of this group had pneumological function tests, however orthopedic operations were only documented in < 5 patients. The stage 3 group had fewer average documented outpatient visits than stage 2, with 9.63 (SD 15.44), and fewer hospital admissions and days.

A large proportion of the stage 4 DMD group had scoliosis (73.02%), cardiomyopathy (49.21%), heart failure (41.27%), and respiratory failure (95.24%), the latter of which was inherent to disease stage algorithm for this group. Dysphagia was documented in 38.10% of the stage 4 group, and over half (55.56%) of the stage 4 group had hypertension, and other disorders of fluid, electrolyte and acid–base balance (52.38%), with (60.32%) prescribed ACE-inhibitors. This group had the most relative outpatient pharmacy prescriptions (15.39 PPY, SD 37.10) compared to less than half of this in stage groups 1 to 3. Ventilation support was used by nearly all of the stage 4 group, which contributed to the use of respiratory management by nearly all stage 4 patients.

Immobility was apparent, with half (49.21%) of the stage 4 group with a paraplegia and tetraplegia diagnosis, decubitus prevention medical aids (49.21%) and laxative prescriptions (50.79%).

The stage 4 group was most likely to be admitted to hospital (95.24%), while outpatient visit HCRU was barely different from that in the stage 3 group. Outpatient pharmacy (15.39 prescriptions PPY, SD 37.10) and medical aids (26.10 prescriptions PPY, SD 73.91 and in 100.00% of the group) however, were prescribed substantially more than in any other stage group, as were physio- or occupational therapy prescriptions (7.84 prescriptions PPY, SD 20.03). Medical aid use consisted mostly of those for mobility, incontinence, disabled vehicle and respiratory therapy.

### Costs

**Fig. 4:**
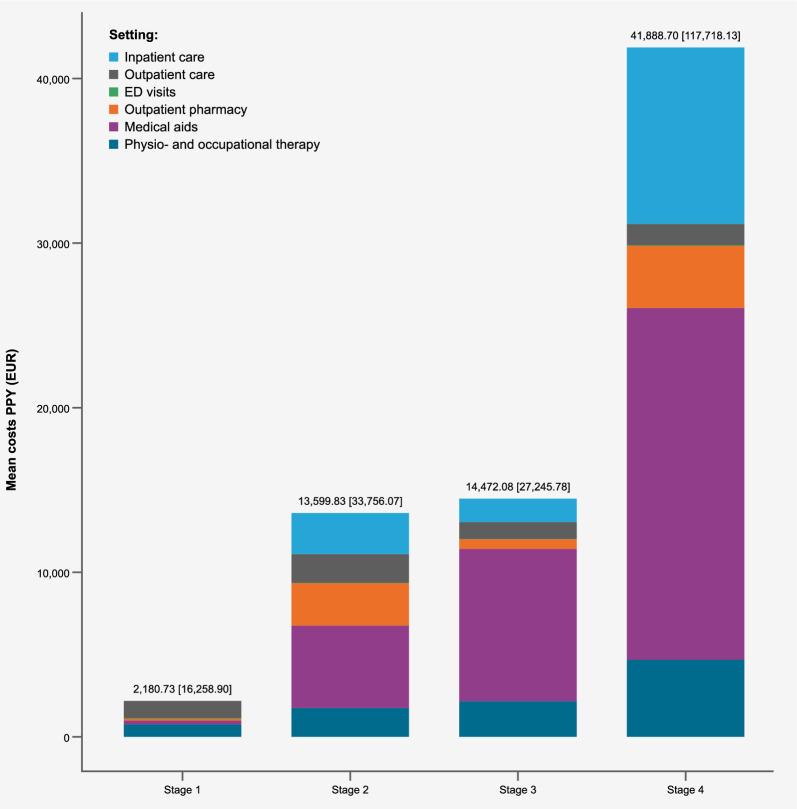
Mean direct healthcare costs (€) by sector and disease stage allocation, per patient year (2017–2021)

Direct medical healthcare costs increased with increasing disease stage; patients in the disease stage 4 incurred nearly 3 times the mean PPY costs of a patient in disease stage groups 2 or 3 (total costs were €41,888.70 (SD €117,718.10) compared to approximately €14,000 (SD between €27,000 and €34,000) for each of stage groups 2 and 3). The difference in costs from stages 2 to 3 were minor (Fig. 4). Costs for medical aids accounted for the highest proportion of costs in all disease stage groups except 1, even accounting for over half the total costs in the disease stage 4 group. Patients in stage 1 incurred a fraction of the costs (€2,180.73), based on the six identified patients throughout the timeframe (Fig. 4).

Inpatient care costs accounted for about a quarter of total medical costs in the disease stage 4 group, substantially higher than in other stage groups (€11,226.56 mean PPY costs) but with an SD of €63,075.10, there was a large variation of inpatient costs in this group (see Supplementary Table 10 for full details on cost data). Patients in stage groups 2 and 3 had on average only about a fifth to half of these inpatient costs (€4,243.64, SD €21,891.10 in stage 2 and €2,508.59, SD €5,915.10 in stage 3). Similarly, outpatient pharmacy costs and physio- and occupational therapy costs were highest in the stage 4 group: €3,832.57 (SD €24,289.59) and €5,027.43 (SD €13,798.80), respectively. Mean costs of respiratory management of the stage 4 patient group were substantially higher than those of other disease stage groups; they generated €38,526.31 mean PPY costs (SD €120,279.10), compared to mean PPY costs of €504.58 (SD €16,812.30) in the stage 3 group.

By age group, mean total direct healthcare costs were low for patients under 8 years, averaging between €4,000.00 and €6,000.00, followed by €22,385.64 (SD €74,866.22) for patients 8–12 years. Mean PPY costs were between €20,000.00 and €30,000.00 among the three older age groups (8–12 years, 13–17 years, and ≥ 18 years). The mean PPY costs were slightly higher among patients 13–17 years than those over 18 years, with €29,745.16 (SD €96,627.09) (see Supplementary Table 11. Among all age groups older than 3 years, the care setting generating the highest share of costs was medical aids. Among age groups older than 7, the settings generating the second and third highest share of costs were inpatient care and outpatient pharmacy, respectively (see Supplementary Fig. 1).

## Discussion

Our adapted algorithm identified 140 males in the database; most patients were allocated to disease stage 4, for whom we found substantially higher direct healthcare costs, especially for medical aids.

### Patient identification

To overcome the challenge of the absence of a DMD-specific ICD-10 code in this large German SHI healthcare claims database, we adapted previously developed algorithms [20–22] and consulted with experts, to approximate a DMD-specific population.

Schrader et al. [20], which contributed to our patient identification algorithm, validated their DMD selection criteria using electronic health records to 74% accuracy with DMD patients [20]. While we could not use a specific DMD/BMD diagnosis code, we adopted elements from their more restrictive narrow definition. This included the criterion of two or more DMD diagnoses, which they found to increase their positive predictive value by around 5 percentage points for all definitions [20]. Requiring patients to have 2 verified outpatient diagnoses at two different clinics resulted in a substantial reduction of our G71.0-diagnosed population (Fig. 1 and Supplementary Table 4), increasing the likelihood that identified patients have a muscular dystrophy (specificity) [22], though we may have incorrectly excluded some muscular dystrophy (possible also some DMD) patients. While we attempted to use age-related mobility criteria to differentiate BMD from DMD patients, one of the age-related criterion was independent of mobility status. Through this criterion, we may have captured patients with other muscle dystrophies [31–33], since 78 male and 15 female patients were 10 years or older and had ventilation, but without mobility status requirements (if they were aged 16 and older) (Supplementary Table 12). Granted, these patients may have also met other age-related criteria, however due to a missing mobility requirement from a certain age (in addition to other age-related criteria), it may have allowed mobile patients that were older to be included.

### Disease stages

An increasing mean age with worsening disease stage reflects the development of DMD progression [34] and is in line with expectations based on age-related criteria in our algorithm. However, we only had a total of 6 patients (out of 134 across the timeframe) that met the criteria for the early ambulatory disease stage, our disease stage 1 group. Iff et al. [27], identified a DMD population of which 38.7% had DMD stage 1; while we based our disease stage criteria heavily on theirs, their DMD patients were selected by use of an additional code that allowed differentiation to BMD patients, which may have made the difference in the proportion by disease stage in their population [27]. They also restricted their patients to ages 2–28, possibly limiting patients in late-stage DMD in their population, where at the same time, we required age-related inclusion criteria, of which the only one applicable to early-stage patients may have been glucocorticoid use between the ages of 4 and 16 years (Fig. 1). Our population was largely comprised of patients in stage 4 group (47.01% met stage 4 criteria at some point), of which 25% were aged 27 or older. Notably, the maximum age in our stage 2 group was 38; since we expect patients to have a median life expectancy of 28.1 years (95% CI 25.1, 30.3) [7], or at least, meet criteria for stage groups 3 and 4 at that age, this indicates that we have captured patients in our DMD population that likely do not have DMD. Further to this point, we had to exclude 24 females who had fulfilled all our other age-related criteria as the final step (Fig. 1). Schrader et al. [20] used an algorithm for a DMD population in Germany, on which we heavily adapted our algorithm, however the distribution by age was substantially different [20]. Using mostly comparable DMD population criteria to those applied to our population, the largest proportion of patients were between the ages of 8 and 14 years (41.4%), and 60.2% were under 14 years of age; in our population only 25% were younger than 13 (in 2021) (Table 2). While a typical DMD phenotype disease course is described in the literature [9–11], there is a spectrum of phenotypes and not all DMD patients follow this typically described course, which can at least partly explain these differences to the literature.

Our population and disease stage algorithms were both based on studies from other countries (USA), based on different data, which likely resulted in a different population distribution in terms of disease heterogeneity, age, and disease stage groups.

### Epidemiology

Extrapolated to the German male population, we found administrative prevalence of DMD to be between 14.85 (95% CI 12.17, 17.95) in 2016 to 18.91 (95% CI 15.70, 22.61) in 2020 per 100,000 males under the age of 40 years. Due to heterogeneous methods, base populations, and prevalence reporting (by total population, by total males, or by males of a particular age group), prevalence rates vary widely and this must be considered when attempting a comparison; a 2020 systematic review estimated the global pooled prevalence to be 7.1 (95% CI 5.0, 10.1) per 100,000 males [13]. Our prevalence rate was per 100,000 males under 40 years of age, more comparable to a rate of 16.8 per 100,000 males under 16 years of age in Sweden (95% CI 11.4, 23.8) [35], and a prevalence of 12.76 (95% CI 8.26, 18.84) per 100,000 males under the age of 20 (1998 in Estonia) [36]. European (France, Germany, Italy, Spain and the United Kingdom) prevalence estimates from 2019 using a system dynamics model based on a triangular distribution, identified incidence rates from the literature and used them to calculate prevalent cases in 2019; the diagnosed prevalence was 6.52 (95% CI 5.04, 8,19) per 100,000 males, and the prevalence among males aged 45 and younger was 12.0 (95% CI 9.2, 15.0) [37]. While methodological differences in prevalence rate calculations make a direct comparison challenging, reported prevalence rates are similar to our findings.

### Burden of disease and HCRU

We found diagnoses, medications, medical aids and procedures indicative of the burden of disease that we would expect with increasing DMD disease stage. Information that emerged from our analysis results that were not a result of our selection or disease stage criteria, reflect characteristics we would expect to see in DMD patients in these disease stages, and the respective HCRU. In the disease stage 2 group, increasing frequency of mobility aids for vehicles, ICD-10 codes for developmental disorders, and diagnosis codes indicating speech development disorders were consistent with published disease progression characteristics [4]. While we did have some patients in this age group far older than we would expect (max age 38), the IQR of these patients was between 7 and 13, pointing to the typical DMD disease course rather than, for example, BMD patients who usually walk normally until the age of 15 years [2]. We also did see most of these patients prescribed occupational or physiotherapy, which continued across the disease stage groups, as recommended [10]. As discussed by Bushby et al. [11] surgical interventions are more likely to be effective before the late ambulatory phase, which we find in our burden of disease analysis of surgical interventions. We observed a higher proportion of long-term therapy with glucocorticoids among patients categorized into the stage 2 group, reflecting that the patients with glucocorticoid therapy typically do not yet display higher disease stage criteria. This particular criterion was based on expert feedback of glucocorticoid treatment as a marker for stage 2 (in the absence of criteria for higher stages). While early ambulatory stage patients still display a few symptoms, initiation of glucocorticoid treatment is recommended during ambulatory phase when motor skills are no longer improving but before a significant physical worsening occurs, carefully balancing the treatment with possible side effects. Diagnoses and medications indicating immobility (constipation diagnosis, laxative prescriptions), and proton-pump inhibitors indicating gastro-esophageal reflux disease as a common side effect of glucocorticoid administration, are also reflective of DMD disease [11], and we saw use increase with increasing disease stage group. The very high use of medical aids in our disease stage 4 group (including decubitus prevention aids and ventilation) reflects the severity of a DMD disease course, especially keeping in mind that we excluded patients over the age of 40 from our study population. Overall, patients had higher HCRU in disease stage 4 group, consistent with Iff et al. [24, 27]. However, while they found increasing average annualized hospital days with each stage, we found stage 2 group to have on average twice the inpatient days as the stage 3 group. While we adapted their disease stage algorithm to a German claims data setting, there are many other factors from differences in the population to the codes used in the algorithm, that could explain the discrepancy in results.

### Costs

As found in previous studies [27, 38, 39] costs increased with increasing disease stage. Although the difference between stage groups 2 and 3 were often very small, costs for the stage 2 group were often higher than the same costs for the stage 3 group. Iff et al. [27] used their disease stage criteria to evaluate costs and found a consistent increase throughout the stages (also from stages 2 to 3), and an even larger magnitude of increase towards stage 4. The latter was also evident in our results, especially for inpatient care, outpatient pharmaceutical prescriptions and medical aid prescriptions.

Similar to the cost patterns observed across disease stages, costs by age group also showed an overall increasing trend. Interestingly, adolescents incurred slightly higher direct healthcare costs than adults. While the stratification by disease stage revealed a distinct cost increase between stages 3 and 4, the age-based analysis showed a more even distribution of costs among patients aged 8 years and older. This aligns with findings in the literature indicating that the economic cost of DMD increases drastically as patients begin to lose ambulation and require more intensive care, often around age 8–12 [6]. Although most patients classified into disease stage groups 3 and 4 were likely to fall within the 13–17 and 18 + age groups (given the age distribution by stage group), the differences in cost patterns between the two stratifications could be reflective of variability in disease progression – some patients may advance more rapidly, while others progress more slowly – for example, due to influence by genetic modifiers and other prognostic factors [40, 41].

To contextualize our findings, we compared our results by disease stage with those reported in previous studies. Iff et al. [27] reported substantially higher total costs for patients across all stages than we found, however, they used slightly different methodology of weighting costs by length of stay [15], and comparability is further limited due to differences in the healthcare system in the US, where direct medical costs of DMD are higher than in Germany, especially due to out-of-pocket payment [39]. As seen with our results in all disease stages, there was high variability in the annual health care costs estimated by Iff et al. as well. Landfeldt et al. [39] collected cost data based on a questionnaire in Germany, and found mean per patient annual direct medical costs in 2012 of around $11,240 international USD (95% CI: $9,720, $14,100), but most of the cost of illness was found to be due to indirect costs, especially loss of quality of life, which amounted to $45,160 international USD. While they were able to consider indirect costs, the total annual cost of illness also increased with increasing disease stage [35].

Medical aids comprised the largest proportion of direct medical costs we observed in the stage 2 group and onwards, a finding also reported by Schreiber-Katz et al. [8]. While standard deviations were large for most of the healthcare costs by sector and by disease stage or age group, they were particularly large in the sector of outpatient pharmacy costs. This suggests that in addition to large variability due to small patient numbers by disease stage, there may be unevenly distributed medication costs. Ataluren (Translarna^®^) is a costly medication available in Europe since 2014; however, there were no documented prescriptions for this medication among the patients included in this study (data not shown).

Since DMD is a childhood onset disease that requires a high level of care from early on, indirect costs due to loss of productivity, including that of parents/caregivers, are estimated to be even higher than the direct medical costs of the illness [6, 39]. Care planning by disease stage is crucial, due to the broad range of needs throughout disease progression.

#### Limitations

We estimated the DMD administrative prevalence in Germany using an approximation of a DMD population, which we did not validate with primary care data. Furthermore, representativeness of the German population can only be guaranteed regarding age and sex. Since DMD is a rare illness (38), representativeness of this disease may be difficult to capture in a sample population. This limitation may influence our results, despite the large database sizes. Regional or socioeconomic influences on DMD prevalence or disease progression and other variables obtained in this study, may not be fully generalizable to the whole German SHI population.

Our algorithm and disease staging were based on coding for healthcare claims purposes. Patients in disease stage group 1 may be underrepresented due to restrictive criteria used in the patient identification algorithm. The low number of patients classified into stage group 1 limits interpretability of burden of disease and direct medical costs. Patients were categorized into the highest disease stage group for which they met the criteria, including patients under 8 years of age, for example if they met the stage 2 criterion of long-term therapy with glucocorticoids. As is the case with other algorithms based on claims data, misidentification and misclassification of patients may occur. Furthermore, patient characteristics related to the employed criteria are informative about the performance of the algorithm and should be interpreted in this context. Diagnoses used to define DMD cases and other outcomes, assume that these codes are accurate, though misclassification of diagnosis and other codes is generally possible. Diagnosis coding accuracy in the outpatient setting may not always reflect the true reason for the visit, or all aspects of it. For example, when a ventilation test is conducted in the inpatient setting, this is coded with an OPS code (which we could observe), however in the outpatient setting these can occur as part of routine visits but may not be coded (OPS codes are only used in the inpatient setting). Also, coding may occur days or even weeks following the outpatient visit, which may be the reason that we see non-specific codes (for example, ICD-10 Z, or −99 codes) that limit the accuracy of the full clinical picture.

A limitation particularly relevant in this rare disease study are the German data protection laws requiring group sizes n < 5 to be reported as such, including extrapolations of this data.

We evaluated data on several healthcare parameters, including ICD-10 codes for diagnoses, ATC codes for prescribed medications, OPS, EBM, and other codes to identify medical aids and allied health services across different healthcare sectors. Some endpoints were not clearly associated with one particular code but could be identified from a few possible codes (for example DMD disease progression stages, where we referenced therapies or diagnoses typical to disease stages) [20, 27]. Furthermore, since hospital stays are documented using a disease-related group code, some documented diagnostic tests and ATC codes may not be visible in our data. In this case, the costs are still available, however it could have affected our pre-defined or burden of disease analysis.

Indirect costs and direct non-healthcare costs associated with DMD were not assessed in the present study due to the inherent limitations of claims data; these aspects are better captured through alternative approaches such as patient or caregiver questionnaires. Finally, while we evaluated HCRU and direct medical costs in patients with DMD, patients likely did not receive a definitive diagnosis immediately upon seeking healthcare for signs and symptoms of DMD, therefore our results are limited to HCRU and direct medical costs that occur following their diagnosis.

## Conclusions

We adapted a DMD patient identification algorithm and disease stage criteria to the German healthcare setting and applied them to a large, representative claims database. This approach enabled a detailed and current picture of the epidemiology, disease burden, HCRU, and direct medical costs associated with DMD in Germany.

## Supplementary Information

Additional file1

Additional file2

## Data Availability

While our epidemiology data is drawn from calculations from a large dataset, the pseudonymized healthcare data was made available to us for the purpose of this project and is stringently protected in a physical location. Due to data protection reasons, it cannot be made available to the public. This is to comply with European General Data Protection Regulation and preserve the individuals’ privacy.

## Abbreviations

ACE: Angiotensin converting enzyme
ATC: Anatomical therapeutic chemical
BMD: Becker muscular dystrophy
CI: Confidence interval
DMD: Duchenne muscular dystrophy
EBM: Einheitlicher Bewertungsmaßstab (German uniform assessment standard)
HCRU: Healthcare resource utilization
ICD-10: International statistical classification of diseases and related health problems (ICD-10)
IQR: Interquartile range
OPS: Operationen- und Prozedurenschlüssel (German procedure classification of operations and procedure)
PPY: Per patient year
SD: Standard deviation
SHI: Statutory health insurance
