# Supplementary material for: Epidemiology, disease burden and costs of Duchenne muscular dystrophy in Germany: an observational, retrospective health claims data analysis
Source: Orphanet J Rare Dis. 2025 Aug 13;20:429. doi: 10.1186/s13023-025-03906-x (PMC12351880; doi:10.1186/s13023-025-03906-x)
Supplement: Supplementary file 1 — Additional file1 [file 13023_2025_3906_MOESM1_ESM.pdf]

Epidemiology, disease burden and costs of Duchenne Muscular Dystrophy in Germany: an observational, retrospective health claims data analysis

Joanna Diesing[1], Janbernd Kirschner[2], Astrid Pechmann[2], Jörg König[3], Leonie Kunk[3], Tarcyane Barata Garcia[1], Carolina Schwedhelm[3], Carsta Militzer-Horstmann[1], Ivonne Hänsel[3], Agnes Kisser[3]

[1]WIG2 GmbH, Scientific Institute for Health Economics and Health System Research, Markt 8, 04109 Leipzig, Germany; [2]Department of Neuropediatrics and Muscle Disorders, Medical Center – University of Freiburg, Faculty of Medicine, Breisacher Str. 62, 79106 Freiburg, Germany; [3]Pfizer Pharma GmbH, Friedrichstraße 110, 10117 Berlin, Germany.

Corresponding author: Carolina Schwedhelm

Supplementary Materials

© 2025 – WIG2 GmbH

Supplemental table 1 Inclusion and exclusion criteria applied to identify DMD patients with sources from which algorithm was adapted

Criteria (inclusion)	Rationale
M2Q of G71.0 diagnosis code (the first year in which this is fulfilled)	ICD-10 code G71.0 used for muscle dystrophies in Germany [1].<br>We implemented M2Q as an internal validation step to minimize possible miscoding [2, 3]
At least 1 intermediate/final diagnosis of G71.0 (M1Q)<br>At least one G71.0 diagnosis in at least two different clinics/hospitals in the first year	Since DMD patient care extends across several specialties [4], these criteria capturing the diagnosis code for muscle dystrophies across medical practices were a further internal validation step [3].
Age < 40	Considering the median life expectancy of DMD patients is 28.1 years (95% CI 25.1, 30.3) [5] or up to early 40s [6], this limit was intended to allow us to capture the full range of DMD stages [2].
At least one of the following age-related additional criteria (fulfilled at least once in the observation period):<br>▪Long-term therapy with glucocorticoids between 4 and 16 years; at least two consecutive quarters of prescription from one of either deflazacort, prednisolone, or prednisone<br>▪Wheelchair use < 16 years of age<br>▪Evidence of walking aid < 13 years of age<br>▪Cardiomyopathy/ACE inhibitors/Beta-blocker prescription and < 26 years of age<br>▪Ventilation ≥ 10 years old	Multiple criteria are considered here to capture patients across the disease course [7]<br>▪Long-term glucocorticoid therapy is normally prescribed to DMD patients (80-85% of patients aged 6-8) but not to patients of other muscle dystrophies [2, 7]<br>▪Independent mobility is typically lost in DMD between the ages of 6 and 13 years [7–9]<br>▪A walking aid with age limitation was intended to different from BMD patients, who typically walk normally until the age of 15 [8, 10, 11]<br>▪Cardiomyopathy/ACE inhibitors/Beta-blocker prescriptions at this age to differentiate DMD patients from other muscular dystrophies, was also used by [7, 12].<br>▪Ventilation at this age was to differentiate DMD patients from patients with other muscular dystrophies (for example, limb girdle) [8, 12]), and from patients who may have a more severe disorder if ventilation is needed before age 10 years [13]. Criterion also used by [2, 7]
Criteria (exclusion)	Rationale
Exclusion of patients receiving ventilation ≤ 3 years of age	It is unlikely in DMD that ventilation would be needed at such an early age and this criterion was used to exclude patients with more severe disorders at this age [7, 14, 15]

Exclusion of patients with no ventilation at the age of ≥30 years of age	DMD patients typically require ventilation by the age of 30; criterion was also used by Schrader et al (2023) [2]
Exclusion of females	DMD is an x-linked disease and was used to exclude females with other muscular dystrophies [7, 14]

Supplementary table 2 Codes used in the algorithm to identify DMD patients in the WIG2 database claims data

**Codes in claims data used to identify DMD patients population**		
**Codes used to determine glucocorticoid therapy**		
**Code type**	Code	Description
**ATC code**	H02AB06	prednisolone
	H02AB07	prednisone
	H02AB13	deflazacort
**Codes used to determine ventilation**		
**Code type**	Code	Description
**ICD-10-GM**	Z99.1	Dependence (long-term) on a respirator
	J96.10	Chronic respiratory failure, not elsewhere classified: Type I
	J96.11	Chronic respiratory failure, not elsewhere classified: Type II
	J96.19	Chronic respiratory insufficiency, not elsewhere classified: Type unspecified
	J96.90	Respiratory insufficiency, unspecified: Type I
	J96.91	Respiratory insufficiency, unspecified: Type II
	J96.99	Respiratory insufficiency, unspecified: Type unspecified
	J95.0	Dysfunction of a tracheostoma
	Z43.0	Supply of a tracheostoma
	Z93.0	Presence of a tracheostoma
**OPS**	5-312	Permanent tracheostomy
	8-711	Mechanical ventilation and respiratory support for newborns and infants
	8-712	Mechanical ventilation and respiratory support in children and adolescents
	8-713	Mechanical ventilation and respiratory support for adults
	8-714	Special procedures for mechanical ventilation in severe respiratory failure
	8-716	Adjustment of home mechanical ventilation
**SHI medical aids/devices**	14.24.01	Aerosol inhalation devices for lower respiratory tract
	14.24.02	Aerosol inhalation devices for upper respiratory tract

	14.24.04	Oxygen therapy devices, pressurized gas filling units	
	14.24.05	Oxygen therapy devices, pressurized and liquid gas	
	14.24.06	Oxygen therapy devices, oxygen concentrators	
	14.24.08	Respiratory therapy for mucus loosening/elimination	
	14.24.09	Respirators for intermittent ventilation up to 30 hPA ventilation pressure	
	14.24.12	Ventilators for life-sustaining ventilation	
	14.24.13	Ventilators for intermittent ventilation with a ventilation pressure > 30 hPA	
	14.24.19	Customized masks for respiratory systems	
	14.24.20	CPAP systems for the treatment of sleep-related breathing disorders	
	14.24.21	Auto-CPAP systems for the treatment of sleep-disordered breathing	
	14.24.22	Bilevel systems for the treatment of sleep-disordered breathing	
	14.24.23	Auto-bilevel CPAP systems for the treatment of sleep-disordered breathing	
	14.24.24	Bilevel CPAP systems for the treatment of sleep-disordered breathing with ST function	
	14.24.25	Special devices for the treatment of sleep-disordered breathing	
**Codes used to determine wheelchair use**			
**Code type**	Code	Description	
**ICD-10-GM**	R26	Reduced mobility	Abnormalities of gait and mobility
	R26.1	Reduced mobility	Paralytic gait
	R26.2	Reduced mobility	Difficulty in walking, not elsewhere classified
	R26.8	Reduced mobility	Other abnormalities of gait and mobility
	R26.81	Reduced mobility	Unsteadiness on feet
	R26.89	Reduced mobility	Other abnormalities of gait and mobility
	R26.9	Reduced mobility	Unspecified abnormalities of gait and mobility
	Z74.09	Reduced mobility	Other reduced mobility
**SHI medical aids/devices**	22.29.01	Mobility assistance, full body, lifting aid	
	22.29.02	Mobility assistance, full body, stand up aid/device for armchair/chairs	
	22.40.01	Mobility assistance, domestic use, Lift, mobile for external operation	
	22.40.02	Mobility assistance, domestic use, Lifter for external operation, wall mounted	
	22.40.04	Mobility assistance, domestic use. Accessories for lift	
	22.40.05	Mobility assistance, domestic use, Stationary lift	
	22.40.06	Mobility assistance, domestic use, ceiling lift	
	22.50.01	Mobility assistance, domestic and outdoor use/road traffic use, ramp system	

	22.50.02	Mobility assistance, domestic and outdoor use/road traffic use, orthopedic scooter
	22.50.03	Mobility assistance, domestic and outdoor use/road traffic use
	22.50.04	Mobility assistance, domestic and outdoor use/road traffic use
	22.50.05	Mobility assistance, domestic and outdoor use/road traffic use
	22.51.01	Mobility assistance, road traffic, two-wheelers for children and adolescents
	22.51.02	Mobility assistance, road traffic, tricycles for children and teenagers
	22.51.03	Mobility assistance, road traffic, accessories for two- / three-wheelers for children and adolescents
	22.51.04	Mobility assistance, road traffic, residual power tricycles for children and adolescents
	22.51.05	Mobility assistance, road traffic, walking bikes
	22.51.99	Mobility assistance, road traffic
	18.46.01	Vehicles for the sick / disabled, indoor, Select shower / toilet wheelchairs
	18.46.02	Vehicles for the sick / disabled, indoor, toilet wheelchairs
	18.46.03	Vehicles for the sick / disabled, indoor, shower wheelchairs
	18.46.04	Vehicles for the sick / disabled, indoor, Wheelchairs with single arm drive
	18.46.05	Vehicles for the sick / disabled, indoor, Indoor power wheelchairs
	18.46.06	Vehicles for the sick / disabled, indoor, power wheelchairs dismountable, foldable for indoor use
	18.46.07	Vehicles for the sick / disabled, indoor, power wheelchairs dismountable, foldable for indoor use
	18.50.01	Vehicles for the sick / disabled, indoor/outdoor/road traffic, Sliding wheelchairs
	18.50.02	Vehicles for the sick / disabled, indoor/outdoor/road traffic, wheelchairs with push rim drive
	18.50.03	Vehicles for the sick / disabled, indoor/outdoor/road traffic, adaptive wheelchairs
	18.50.04	Vehicles for the sick / disabled, indoor/outdoor/road traffic, Mobile walking aids, in-door and outdoor power wheelchairs
	18.50.05	Vehicles for the sick / disabled, indoor/outdoor/road traffic, Mobile walking aids with wheelchair function, Select power wheelchairs for children and adolescents
	18.50.06	(planned product subgroup: power wheelchairs dismountable, foldable for indoor use)
	18.50.07	Vehicles for the sick / disabled, indoor/outdoor/road traffic, Wheelchairs with fixed, residual crane-assisting drive, power wheelchairs dismountable, foldable for indoor and outdoor use / road transport select
	18.51.01	Vehicles for the sick / disabled, road traffic, Electric wheelchairs for outdoor use

	18.51.02	Vehicles for the sick / disabled, road traffic, Electric scooters
	18.51.03	Vehicles for the sick / disabled, road traffic
	18.51.04	Vehicles for the sick / disabled, road traffic, Hand crank drive pretensioned/hang-in bicycles for children and teenagers
	18.51.05	Vehicles for the sick / disabled, road traffic, Electromobiles
	18.65.01	Vehicles for the sick / disabled, stairs, staircase vehicle
	18.65.02	Vehicles for the sick / disabled, stairs
	18.65.03	Vehicles for the sick / disabled, stairs
	18.65.04	Vehicles for the sick / disabled, stairs
	18.65.05	Vehicles for the sick / disabled, stairs
	18.65.06	Vehicles for the sick / disabled, stairs
	18.65.07	Vehicles for the sick / disabled, stairs
	18.65.08	Vehicles for the sick / disabled, stairs
	18.65.09	Vehicles for the sick / disabled, stairs
	18.65.10	Vehicles for the sick / disabled, stairs
	18.65.11	Vehicles for the sick / disabled, stairs
	18.65.12	Vehicles for the sick / disabled, stairs
	18.65.13	Vehicles for the sick / disabled, stairs
	18.65.99	Vehicles for the sick / disabled, stairs
**Codes used to determine use of mobility assistance**		
**Code type**	Code	Description
**ICD-10-GM**	Z99.3	Dependence (long-term) on assistive devices, medical equipment, or supplies, not elsewhere classified.
	Z46.7	Providing with and fitting other medical devices or aids
	Z46.8	Providing and fitting an unspecified medical device or appliance.
	Z46.9	Providing and fitting an unspecified medical device or appliance
**SHI medical aids/devices**	10.46.01	Walking aid, indoors, Walking frames
	10.46.02	Walking aid, indoors, Walking frames
	10.46.04	Walking aid, indoors, Mobile walking frames with forearm supports
	10.50.04	Walking aid, indoors/outdoors/street traffic, Mobile walking aids
	10.50.05	Walking aid, indoors/outdoors/street traffic, Mobile walking aids with wheelchair function
**Codes used to determine cardiomyopathy**		

Code type	Code	Description
**ICD-10-GM**	I42	Cardiomyopathy (all)
**ATC**	C09A	ACE inhibitors or angiotensin
	C09B	ACE inhibitors or angiotensin
	C07A	Beta Blocking Agents
	C09C	Angiotensin II receptor blockers

Supplementary table 3 Codes (by category) used to determine disease stage allocation and pre-defined healthcare resource use

Disease/ Treatment	Source of healthcare data					
	ICD	EBM	DRG	OPS	SHI medical aids/devices	ATC Codes
**Codes for approximation of DMD disease stage 1: Early ambulatory**						
**No codes defined**						
**Codes for approximation of DMD disease stage 2: Late ambulatory**						
**Long-term therapy with glucocorticoids (prednisolone, prednisone or deflazacort)**						H02AB06<br>H02AB07<br>H02AB13
**Scooter or wheelchair prescription (manual) or walking aid assistance**	Z99.3;<br>Z46.7;<br>Z46.8;<br>Z46.9;				Manual<br>18.50.02;<br>18.50.03;<br>18.51.01;<br>18.51.04;<br>10.46.01;<br>10.46.02<br>10.46.04;<br>10.50.01;<br>10.50.02;<br>10.50.03;<br>10.50.04;<br>10.50.05<br>22.50.02<br>22.50.03<br>22.50.04<br>22.50.05<br>22.51.01<br>22.51.02<br>22.51.03<br>22.51.04<br>22.51.05<br>22.51.99	
**Orthotic or prosthetic therapy (footplate, ankle motion, inner boot)**					24.01<br>24.03<br>31.03	

**ACE/ARB, Beta-Blocker prescription or cardiomy-opathy diagnosis**	I42					C09A;<br>C09B;<br>C09C;<br>C01CX09<br>C07A
**Codes for approximation of DMD disease stage 3: Early non-ambulatory**						
**Powered wheelchair**					18.46.05;<br>18.50.04;<br>18.50.05;<br>18.50.06;<br>18.51.02;<br>18.51.05;<br>18.46.04<br>18.46.06<br>18.46.07	
**Mobility**					22.29.01<br>22.29.02<br>22.40.01<br>22.40.02<br>22.40.04<br>22.40.05<br>22.40.06<br>22.50.01<br>18.46.01<br>18.46.02<br>18.46.03<br>18.50.01<br>18.50.07<br>18.51.03<br>18.65.01<br>18.65.02<br>18.65.03<br>18.65.04<br>18.65.05<br>18.65.06<br>18.65.07<br>18.65.08<br>18.65.09<br>18.65.10<br>18.65.11<br>18.65.12<br>18.65.13<br>18.65.99	

**Pulmonary management; nasal-, positive airway and breathing devices**					14.24.01<br>14.24.02<br>14.24.04<br>14.24.05<br>14.24.06<br>14.24.19<br>14.24.20<br>14.24.21<br>14.24.22<br>14.24.23<br>14.24.24<br>14.24.25	
**Scoliosis therapy**	M41			5-83X	23.15	
**Hospital bed or mattress**					50.45.01<br>50.45.02<br>50.45.03<br>50.45.04<br>50.45.06	
**ACE/ARB, Beta-Blocker prescription or cardiomy-opathy diagnosis**	I42					C09A;<br>C09B;<br>C09C;<br>C01CX09<br>C07A
**Codes for approximation of DMD disease stage 4: Late non-ambulatory**						
**Defibrillator, ventricular support, heart transplant**				5-375<br>5-377<br>5-378		
**Tracheostomy and rela-ted products**	Z93.0<br>Z43.0<br>J95.0				12.24	
**Ventilation support and related devices**	J96.10<br>J96.11<br>J96.19<br>J96.90<br>J96.91<br>J96.99<br>Z99.1			5-312;<br>8-711<br>8-712;<br>8-713;<br>8-714<br>8-716	14.24.09<br>14.24.12<br>14.24.13	
**Enteral formula, nutri-tion infusion pump**				8-017	03.36	
**PEG/PEJ tube**	T85.74			5-445<br>5-449.n		
**Pre-defined variables**						
**Ataluren (Translarna®)**						M09AX03
**Scoliosis**	M41			5-83X	23.15	

© 2025 – WIG2 GmbH

**PEG/PEJ tube**	T85.74			5-445<br>5-449.n		
**Respiratory management**						
**Codes used to identify respiratory management costs**	Z99.1			5-312	14.24.01	
	J96.10			8-711	14.24.02	
	J96.11			8-712	14.24.04	
	J96.19			8-713	14.24.05	
	J96.90			8-714	14.24.06	
	J96.91			8-716	14.24.08	
	J96.99				14.24.09	
	J95.0				14.24.12	
	Z43.0				14.24.13	
	Z93.0				14.24.19	
					14.24.20	
					14.24.21	
					14.24.22	
					14.24.23	
					14.24.24	
					14.24.25	
**Cardiac management**						
**Codes used to identify cardiac management costs**	I42					C09A
						C09B
						C07A
						C09C
						C01CX09

Supplementary table 4 The number of patients (N) included in the DMD study population in total by year, by inclusion/exclusion step

	First G71.0, M2Q (first year in which this is fulfilled)						
**Criteria**	Patients (N)	2016	2017	2018	2019	2020	2021
**G71.0, M2Q (first year in which this is fulfilled)**	2,517	1,264	366	231	242	206	208
**At least 1 "intermediate/final diagnosis" G71.0-M1Q***	2,062	1,117	284	164	157	132	208
**At least one G71.0 diagnosis in at least two different clinics/hospitals in the first year**	662	423	69	44	45	33	48
**Age < 40**	317	214	29	15	14	18	27
**Exclusion of patients receiving ventilation ≤ 3 years of age**	315	213	29	15	13	18	27
**At least one of the following age-related additional criterion (fulfilled in at least one year of age)**	165	123	11	6	7	10	8

**Long-term therapy with glucocorticoids between 4 and 16 years; at least two consecutive quarters of prescription from one of either deflazacort, prednisolone, or prednisone**	35	25	< 5	< 5	< 5	< 5	< 5
**Wheelchair use < 16 years of age**	66	43	7	< 5	< 5	6	< 5
**Evidence of walking aid < 13 years of age**	21	13	< 5	< 5	< 5	< 5	< 5
**Cardiomyopathy/ACE inhibitors/Beta-blocker prescription < 26 years of age**	77	63	< 5	< 5	< 5	< 5	< 5
**Ventilation ≥ 10 years old**	93	80	5	< 5	< 5	< 5	< 5
**Exclusion of patients with no ventilation at the age of ≥30 years of age**	164	122	11	6	7	10	8
**Exclusion of females**	140	108	9	6	< 5	9	<5
**At least 12 months of continuous observation data following the first G71.0-M2Q diagnosis**	134	107	9	6	< 5	8	0

Supplementary table 5 Longitudinal analysis of the most frequently documented 3-digit ICD-10 GM codes from 2017–2021, by DMD disease stage approximation

ICD code	Description	Stage 1	Stage 2	Stage 3	Stage 4
**Z00**	General examination and investigation of persons without complaint and reported diagnosis	5 (83.33%)			
**Z01**	Other special examinations and investigations of persons without complaint or reported diagnosis		25 (55.56%)	19 (38.00%)	36 (57.14%)
**H52**	Disorders of refraction and accommodation	*	24 (53.33%)	11 (22.00%)	
**Z25**	Need for immunization against other single viral diseases	*	20 (44.44%)	20 (40.00%)	33 (52.38%)
**J06**	Acute upper respiratory infections of multiple and unspecified sites	*	19 (42.22%)	22 (44.00%)	37 (58.73%)
**F82**	Specific developmental disorder of motor function	*	19 (42.22%)	13 (26.00%)	
**F83**	Mixed specific developmental disorders	*	19 (42.22%)		
**F80**	Specific speech articulation disorder	*	19 (42.22%)		
**Z27**	Need for immunization against combinations of infectious diseases		19 (42.22%)		

**Q66**	Congenital deformities of feet		15 (33.33%)	16 (32.00%)	
**A09**	Other gastroenteritis and colitis of infectious and unspecified origin	*	14 (31.11%)		
**Z11**	Special screening examination for infectious and parasitic diseases		13 (28.89%)		36 (57.14%)
**U99**	Unassigned key number		13 (28.89%)		35 (55.56%)
**E55**	Vitamin D deficiency		13 (28.89%)		
**H50**	Other strabismus		13 (28.89%)		
**M21**	Other acquired deformities of limbs		12 (26.67%)		
**Z99**	Dependence on enabling machines and devices, not elsewhere classified		11 (24.44%)	19 (38.00%)	59 (93.65%)
**M62**	Other disorders of muscle		11 (24.44%)	12 (24.00%)	31 (49.21%)
**M24**	Other specific joint derangements		11 (24.44%)	12 (24.00%)	
**R26**	Abnormalities of gait and mobility		11 (24.44%)	11 (22.00%)	24 (38.1%)
**H10**	Conjunctivitis	*	11 (24.44%)		
**M41**	Scoliosis			19 (38.00%)	46 (73.02%)
**I42**	Cardiomyopathy			14 (28.00%)	31 (49.21%)
**K59**	Constipation			12 (24.00%)	36 (57.14%)
**Z26**	Need for immunization against other single infectious diseases	*		12 (24.00%)	
**E66**	Obesity			12 (24.00%)	
**G47**	Sleep disorders			11 (22.00%)	24 (38.1%)

**I50**	Heart failure			10 (20.00%)	26 (41.27%)
**F43**	Reaction to severe stress, and adjustment disorders			10 (20.00%)	
**Z76**	Persons encountering health services in other circumstances			10 (20.00%)	
**R11**	Nausea and vomiting			10 (20.00%)	
**J96**	Respiratory failure, not elsewhere classified				60 (95.24%)
**Z74**	Problems related to care-provider dependency				43 (68.25%)
**I10**	Essential (primary) hypertension				35 (55.56%)
**E87**	Other disorders of fluid, electrolyte, and acid-base balance				33 (52.38%)
**G82**	Paraplegia and tetraplegia				31 (49.21%)
**R06**	Abnormalities of breathing				26 (41.27%)
**R13**	Dysphagia				24 (38.10%)

*Note: diagnosis codes for which < 5 patients (n) were documented as 20 most frequent were not reported, however codes for which a number could be reported in other stages, and it was recorded in < 5 in other stages were marked with an * when these were under the 20 most frequent codes for patients of other stages

Supplementary table 6: Longitudinal analysis of the most frequently documented 5-digit ATC codes from 2017–2021, by DMD disease stage approximation

5-digit ATC code	Medications	Stage 1	Stage 2	Stage 3	Stage 4
**A11CC**	Vitamin D and analogs	*	31 (68.89%)	18 (36.00%)	22 (34.92%)
**H02AB**	Glucocorticoids	*	31 (68.89%)	12 (24.00%)	
**M01AE**	Propionic acid derivatives	*	22 (48.89%)	18 (36.00%)	27 (42.86%)
**R01AA**	Sympathomimetics, pure	*	17 (37.78%)	8 (16.00%)	
**A12AA**	Calcium		11 (24.44%)		
**R05CP**	Herbal expectorants	*	10 (22.22%)		
**C09AA**	ACE inhibitors, pure		9 (20.00%)	22 (44.00%)	38 (60.32%)

**A06AD**	Osmotic laxatives	*	7 (15.56%)	12 (24.00%)	32 (50.79%)
**N02BE**	Anilide	*	7 (15.56%)		
**B05BB**	Solutions with an effect on the electrolyte balance		5 (11.11%)	7 (14.00%)	26 (41.27%)
**N02BB**	Pyrazolones		5 (11.11%)	6 (12.00%)	30 (47.62%)
**R05CB**	Mucolytics	*	5 (11.11%)	5 (10.00%)	13 (20.63%)
**R05DB**	Other antitussives	*	5 (11.11%)		
**D06AX**	Other antibiotics for topical use		*	6 (12.00%)	
**J01CA**	Penicillins with extended spectrum of activity		*	5 (10.00%)	17 (26.98%)
**C07AB**	Beta-adrenoceptor antagonists, selective			18 (36.00%)	34 (53.97%)
**A02BC**	Proton pump inhibitors			11 (22.00%)	27 (42.86%)
**R03AC**	Salbutamol-ratiopharm;Sultanol;Berotec;SalbuHEXAL;Salbutamol AL			8 (16.00%)	17 (26.98%)
**B01AB**	Selective beta2-adrenoceptor agonists			8 (16.00%)	
**J01FA**	Macrolides			6 (12.00%)	11 (17.46%)
**J01MA**	Fluoroquinolones			5 (10.00%)	
**J01CR**	Combinations of penicillins, incl. beta-lactamase inhibitors			*	21 (33.33%)
**J01DC**	2nd generation cephalosporins	*			16 (25.4%)
**R04AX**	Other inhalants				13 (20.63%)
**C03CA**	Sulfonamides, pure				13 (20.63%)
**D07AC**	Corticosteroids, highly effective (group III)				13 (20.63%)
**S01CA**	Corticosteroids and anti-infectives in combination				11 (17.46%)
**C03DA**	Aldosterone antagonists				11 (17.46%)
**D01AC**	Imidazole and triazole derivatives				11 (17.46%)

*Note: medications for which <5 patients (n) were documented as 20 most frequent were not reported, however those medications for which a number could be reported in other stages, and it was recorded in <5 in other stages were marked with an * when these were under the 20 most frequent medications for patients of other stages

Supplementary table 7: Longitudinal analysis of the most frequently documented 4-digit SHI medical aid codes from 2017–2021, by DMD disease stage approximation

SHI Code	Medical Aid English	Stage 1	Stage 2	Stage 3	Stage 4
**1899**	Patient/disabled vehicle - Without special application location/additions		27 (60.00%)	34 (68.00%)	56 (88.89%)

**1850**	Patient/disabled vehicle - Interior and exterior/road traffic		26 (57.78%)	26 (52.00%)	39 (61.90%)
**2303**	Orthoses/splints - Foot	*	20 (44.44%)	10 (20.00%)	
**803**	Insoles - Foot	*	14 (31.11%)	5 (10.00%)	
**3103**	Shoes - Foot	*	12 (26.67%)	11 (22.00%)	12 (19.05%)
**2399**	Orthoses/splints - Without special application site/additions		11 (24.44%)		
**2521**	Visual aids - Eye/vision		9 (20.00%)	6 (12.00%)	
**2251**	Mobility aids - Road traffic		9 (20.00%)		
**2611**	Seating aids - Body/trunk		8 (17.78%)	14 (28.00%)	26 (41.27%)
**440**	Bathing and showering aids - Domestic use		6 (13.33%)	12 (24.00%)	12 (19.05%)
**1050**	Walking aids - Indoor and outdoor/road traffic		6 (13.33%)		
**2302**	Orthoses/splints - Ankle		5 (11.11%)		
**2699**	Seating aids - Without special application site/additions		*	11 (22.00%)	15 (23.81%)
**1940**	Nursing articles - Domestic use		*	23 (46.00%)	44 (69.84%)
**1424**	Inhalation/respiratory therapy devices - Respiratory organs		*	18 (36.00%)	61 (96.83%)
**1599**	Incontinence aids - Without special application site/additions		*	11 (22.00%)	31 (49.21%)
**899**	Insoles - Without special application site/additions		*	5 (10.00%)	
**2240**	Mobility aids - Domestic area			13 (26.00%)	36 (57.14%)
**3340**	Toilet aids - Domestic use			11 (22.00%)	16 (25.40%)
**1129**	HiMi against decubitus - Whole body			8 (16.00%)	31 (49.21%)
**1499**	Inhalation/respiratory therapy devices - Without special application site/additions			6 (12.00%)	48 (76.19%)
**2229**	Mobility aids - Whole body			5 (10.00%)	
**2307**	Orthoses/splints - Hand			5 (10.00%)	
**1846**	Patient/disabled vehicle - Interior			*	14 (22.22%)
**2130**	Measuring devices for body conditions - Skin				24 (38.10%)
**1139**	HiMi against decubitus - Buttocks				20 (31.75%)

**124**	Suction devices - Respiratory organs				18 (28.57%)
**1525**	Incontinence aids - Urinary/digestive organs				15 (23.81%)
**399**	Application aids - Without special application site/additions				15 (23.81%)
**2299**	Mobility aids - Without special application site/additions				14 (22.22%)

*Note: medical aids for which <5 patients (n) were documented as 20 most frequent were not reported, however patients for whom medical aids were documented that were also recorded under the 20 most frequent medications for which ≥5 patients were recorded in other disease stages, was marked with an *

Supplementary table 8: Longitudinal analysis of the most frequently documented operations/procedures, by 3-digit OPS codes from 2017–2021, by DMD disease stage approximation

Most frequent 20 documented operations/procedures (by OPS code) from 2017-2021, by DMD disease stage allocation

OPS Code	Operation/Procedure English	Stage 2	Stage 3	Stage 4
**9-98**	Need for care and day-care pediatric treatment	14 (31.11%)	15 (30.00%)	52 (82.54%)
**1-79**	Physiological function tests	11 (24.44%)	15 (30.00%)	29 (46.03%)
**1-71**	Pneumological functional examinations	8 (17.78%)	9 (18.00%)	26 (41.27%)
**5-85**	Operations on muscles, tendons, fasciae, and bursae	7 (15.56%)	*	
**8-31**	Immobilization with plaster cast	5 (11.11%)		
**8-93**	Monitoring of respiration, heart, and circulation	*	9 (18.00%)	43 (68.25%)
**9-40**	Psychosocial, psychosomatic, and neuropsychological therapy	*	*	9 (14.29%)
**3-99**	Additional information on imaging procedures	*	8 (16.00%)	9 (14.29%)
**8-83**	Therapeutic catheterization and cannulation of vessels	*	*	15 (23.81%)
**8-80**	Transfusion of blood cells	*	*	9 (14.29%)
**8-90**	Anesthesia	*		8 (12.7%)
**8-98**	Other multimodal complex treatment		*	31 (49.21%)
**3-20**	Computed tomography [CT], native		*	13 (20.63%)
**3-22**	Computed tomography [CT] with contrast medium		*	12 (19.05%)
**8-39**	Positioning treatment		*	9 (14.29%)
**8-71**	Mechanical ventilation and respiratory support via mask or tube and ventilation weaning			51 (80.95%)
**8-70**	Access for mechanical ventilation and measures to keep the airways open			31 (49.21%)
**1-62**	Diagnostic tracheobronchoscopy			16 (25.4%)

**1-63**	Diagnostic endoscopy of the upper digestive tract			10 (15.87%)
**1-61**	Diagnostic endoscopy of the upper respiratory tract			10 (15.87%)
**1-84**	Diagnostic puncture and aspiration			8 (12.70%)
**1-20**	Neurological examinations			7 (11.11%)

*Note: codes for which < 5 patients (n) were documented as 20 most frequent were not reported, however codes for which a number could be reported in other stages, and it was recorded in < 5 in other stages were marked with an * when these were under the 20 most frequent codes for patients of other stages

No OPS codes were documented for patients allocated to DMD stage 1

Supplementary table 9 Mean number of HCRU by sector per patient year (PPY), among all patients and among patients with at least 1 HCRU, stratified by disease stage and across the timeframe 2017–2021

Sector	DMD stage (n)	Mean HCRU PPY (SD) - All patients	Patients ≥ 1 HCRU (%)	Mean HCRU PPY (SD) - Patients with ≥1 HCRU
**Inpatient Days**	Stage 1 (n=6)	- (-)	0 (0.00%)	0 (-)
	Stage 2 (n=45)	2.80 (19.82)	24 (53.33%)	5.11 (26.48)
	Stage 3 (n=50)	1.39 (4.26)	24 (48.00%)	2.47 (4.83)
	Stage 4 (n=63)	7.22 (21.70)	60 (95.24%)	7.56 (21.70)
**Hospital admissions**	Stage 1 (n=6)	- (-)	< 5 (-)	- (-)
	Stage 2 (n=45)	0.63 (2.63)	26 (57.78%)	1.05 (3.29)
	Stage 3 (n=50)	0.38 (0.92)	26 (52.00%)	0.64 (0.97)
	Stage 4 (n=63)	1.66 (3.66)	60 (95.24%)	1.74 (3.60)
**Outpatient visits**	Stage 1 (n=6)	8.91 (11.88)	6 (100.00%)	8.91 (11.88)
	Stage 2 (n=45)	12.69 (20.59)	45 (100.00%)	12.69 (20.59)
	Stage 3 (n=50)	9.63 (15.44)	50 (100.00%)	9.63 (15.44)
	Stage 4 (n=63)	10.46 (19.07)	63 (100.00%)	10.46 (19.07)
**ED visits**	Stage 1 (n=6)	- (-)	< 5 (-)	- (-)
	Stage 2 (n=45)	0.24 (0.73)	16 (35.56%)	0.58 (0.99)
	Stage 3 (n=50)	0.24 (0.66)	21 (42.00%)	0.47 (0.79)
	Stage 4 (n=63)	0.42 (1.24)	39 (61.90%)	0.68 (1.47)
**Number of prescriptions (outpatient pharmacy)**	Stage 1 (n=6)	6.82 (13.93)	6 (100.00%)	6.82 (13.91)
	Stage 2 (n=45)	8.06 (15.84)	41 (91.11%)	8.31 (15.89)
	Stage 3 (n=50)	7.02 (12.49)	49 (98.00%)	7.06 (12.43)
	Stage 4 (n=63)	15.39 (37.10)	62 (98.41%)	15.58 (37.23)
**Number of cases**	Stage 1 (n=6)	2.36 (4.47)	5 (83.33%)	3.25 (4.87)

© 2025 – WIG2 GmbH

(Physio- and occupational therapy)	Stage 2 (n=45)	4.65 (7.26)	42 (93.33%)	4.80 (7.11)
	Stage 3 (n=50)	5.06 (10.47)	42 (84.00%)	5.75 (10.87)
	Stage 4 (n=63)	7.84 (20.03)	58 (92.06%)	8.41 (20.55)
Number of prescriptions (SHI medical aids)	Stage 1 (n=6)	- (-)	< 5 (-)	- (-)
	Stage 2 (n=45)	3.63 (7.64)	43 (95.56%)	3.74 (7.70)
	Stage 3 (n=50)	6.16 (10.92)	44 (88.00%)	7.0 (11.08)
	Stage 4 (n=63)	26.10 (73.91)	63 (100.00%)	26.10 (73.63)

Abbreviations: DMD: Duchenne muscular dystrophy; **ED:** emergency department; **HCRU:** healthcare resource use; **PPY** per patient year; **SD**: standard deviation.

Supplementary table 10 Mean PPY costs by sector (€, with SD) among all patients and among patients with at least 1 HCRU, stratified by disease stage and across the timeframe 2017–2021

Sector	DMD stage (n)	Mean costs in € PPY (SD) - All patients	Patients ≥1 HCRU (%)	Mean costs in € PPY (SD) - Patients with ≥1 HCRU
Inpatient costs	Stage 1 (n=6)	- (-)	<5 (-)	- (-)
	Stage 2 (n=45)	2,501.28 (17,107.62)	24 (53.33%)	4,243.64 (21,891.10)
	Stage 3 (n=50)	1,428.44 (5,154.38)	24 (48.00%)	2,508.59 (5,915.14)
	Stage 4 (n=63)	10,719.94 (61,962.38)	60 (95.24%)	11,226.56 (63,075.13)
Outpatient costs	Stage 1 (n=6)	1,039.58 (1,455.09)	6 (100.00%)	1,039.58 (1,455.09)
	Stage 2 (n=45)	1,746.56 (3,298.89)	45 (100.00%)	1,746.56 (3,298.89)
	Stage 3 (n=50)	1,032.33 (1,818.11)	50 (100.00%)	1,032.33 (1,818.11)
	Stage 4 (n=63)	1,291.77 (2,759.27)	63 (100.00%)	1,291.77 (2,759.27)
ED visits costs	Stage 1 (n=6)	- (-)	<5 (-)	- (-)
	Stage 2 (n=45)	10.26 (39.26)	16 (35.56%)	24.84 (54.56)
	Stage 3 (n=50)	9.95 (28.32)	21 (42.00%)	19.78 (32.34)
	Stage 4 (n=63)	30.37 (119.00)	39 (61.90%)	49.35 (146.76)
Outpatient pharmacy costs	Stage 1 (n=6)	123.91 (1,629.01)	6 (100.00%)	123.91 (1,673.00)
	Stage 2 (n=45)	2,577.45 (22,189.92)	41 (91.11%)	2,658.93 (22,528.86)
	Stage 3 (n=50)	591.08 (1,678.10)	49 (98.00%)	595.01 (1,679.40)
	Stage 4 (n=63)	3,785.82 (24,149.38)	62 (98.41%)	3,832.57 (24,289.59)
Physio- and occupational therapy costs	Stage 1 (n=6)	763.15 (1,998.11)	5 (83.33%)	1,049.23 (2,200.72)
	Stage 2 (n=45)	1,757.34 (2,734.19)	42 (93.33%)	1,812.93 (2,659.67)
	Stage 3 (n=50)	2,149.06 (4,590.37)	42 (84.00%)	2,439.94 (4,750.22)

© 2025 – WIG2 GmbH

	Stage 4 (n=63)	4,686.42 (13,411.42)	58 (92.06%)	5,027.43 (13,798.80)
**SHI medical aids costs**	Stage 1 (n=6)	- (-)	<5 (-)	- (-)
	Stage 2 (n=45)	5,006.94 (9,434.04)	43 (95.56%)	5,165.33 (9,387.39)
	Stage 3 (n=50)	9,261.22 (21,117.64)	44 (88.00%)	10,514.71 (22,098.56)
	Stage 4 (n=63)	21,374.37 (47,346.29)	63 (100.00%)	21,374.37 (47,022.27)
**Cardiac management costs**	Stage 1 (n=6)	- (-)	<5 (-)	- (-)
	Stage 2 (n=45)	36.32 (628.20)	13 (28.89%)	126.72 (982.42)
	Stage 3 (n=50)	1,422.77 (14,669.84)	32 (64.00%)	2,064.71 (17,592.19)
	Stage 4 (n=63)	1,673.17 (21,970.58)	47 (74.60%)	2,226.16 (25,281.12)
**Respiratory management costs**	Stage 1 (n=6)	- (-)	<5 (-)	- (-)
	Stage 2 (n=45)	- (-)	<5 (-)	- (-)
	Stage 3 (n=50)	146.88 (9,455.75)	13 (26.00%)	504.58 (16,812.35)
	Stage 4 (n=63)	38,482.11 (124,361.79)	62 (98.41%)	38,526.31 (120,279.06)
**Total costs (€)***	Stage 1 (n=6)	2,180.73 (16,258.90)	6 (100.00%)	2,180.73 (16,258.90)
	Stage 2 (n=45)	13,599.83 (33,756.07)	45 (100.00%)	13,599.83 (33,756.07)
	Stage 3 (n=50)	14,472.08 (27,245.78)	50 (100.00%)	14,472.08 (27,245.78)
	Stage 4 (n=63)	41,888.70 (117,718.13)	63 (100.00%)	41,888.70 (117,718.13)

Abbreviations: DMD: Duchenne muscular dystrophy**; ED:** emergency department**; HCRU:** healthcare resource use**; PPY** per patient year; **SD**: standard deviation.

Supplementary table 11 Mean PPY costs by sector (€, with SD) among all patients and among patients with at least 1 HCRU, stratified by age group and across the timeframe 2017–2021

Sector	Age group, years (n)	Mean costs in € PPY (SD) - All patients	Patients ≥1 HCRU (%)	Mean costs in € PPY (SD) - Patients with ≥1 HCRU
**Inpatient costs**	00-03 (n=6)	- (-)	<5 (-)	- (-)
	04-07 (n=26)	528.59 (3,584.18)	10 (38.46%)	1,287.68 (3,635)
	08-12 (n=43)	7,126.16 (62,130.06)	22 (51.16%)	11,532.31 (78,772.29)
	13-17 (n=54)	7,870.95 (46,966.73)	36 (66.67%)	11,206.96 (55,717.25)
	≥18 (n=79)	5,466.32 (28,891.14)	66 (83.54%)	6,205.79 (30,503.03)
**Outpatient costs**	00-03 (n=6)	1,496.59 (944.1)	6 (100.00%)	1,496.59 (944.1)
	04-07 (n=26)	1,406.15 (1,802.57)	26 (100.00%)	1,406.15 (1,802.57)
	08-12 (n=43)	1,790.08 (3,400.96)	43 (100.00%)	1,790.08 (3,400.96)
	13-17 (n=54)	1,394.7 (2,312.51)	54 (100.00%)	1,394.7 (2,312.51)

	≥18 (n=79)	1,127.47 (2,663.23)	79 (100.00%)	1,127.47 (2,663.23)
**ED visits costs**	00-03 (n=6)	- (-)	<5 (-)	- (-)
	04-07 (n=26)	15.2 (54.53)	9 (34.62%)	34.16 (74.54)
	08-12 (n=43)	8.96 (24.78)	19 (44.19%)	18.19 (27.55)
	13-17 (n=54)	21.17 (75.64)	20 (37.04%)	48.07 (106.87)
	≥18 (n=79)	28.7 (125.13)	45 (56.96%)	47.33 (156.07)
**Outpatient pharmacy costs**	00-03 (n=6)	79.65 (2,194.45)	6 (100.00%)	79.65 (2218.73)
	04-07 (n=26)	193.45 (1,551.38)	25 (96.15%)	197.12 (1542.64)
	08-12 (n=43)	2,942.51 (26,314.86)	43 (100.00%)	2,942.51 (26312.64)
	13-17 (n=54)	5,944.41 (37,986.92)	51 (94.44%)	6,118.27 (38532.31)
	≥18 (n=79)	1,706.25 (7,145.65)	77 (97.47%)	1,731.49 (7,190.5)
**Physio- and occupational therapy costs**	00-03 (n=6)	- (-)	<5 (-)	- (-)
	04-07 (n=26)	1,538.48 (2,177.4)	24 (92.31%)	1,593.35 (2,103.82)
	08-12 (n=43)	1,906.41 (3,346.68)	40 (93.02%)	2,004.05 (3,323.4)
	13-17 (n=54)	2,393.2 (7,095.76)	46 (85.19%)	2,662.31 (7,391.11)
	≥18 (n=79)	3,799.32 (11,545.88)	70 (88.61%)	4,241.66 (12,111.12)
**SHI medical aids costs**	00-03 (n=6)	- (-)	<5 (-)	- (-)
	04-07 (n=26)	2,206.64 (6,241.55)	19 (73.08%)	2,741.15 (5,515.83)
	08-12 (n=43)	8,611.51 (17,620.55)	36 (83.72%)	9,693.53 (18,137.55)
	13-17 (n=54)	12,120.73 (30,660.27)	48 (88.89%)	13,558.85 (32,054.16)
	≥18 (n=79)	15,388.02 (39,772.16)	76 (96.20%)	15,882.2 (40,081.22)
**Cardiac management costs**	00-03 (n=6)	- (-)	<5 (-)	- (-)
	04-07 (n=26)	- (-)	<5 (-)	- (-)
	08-12 (n=43)	82.23 (708.19)	13 (30.23%)	215.63 (925.19)
	13-17 (n=54)	137.65 (680.46)	28 (51.85%)	254.39 (891.49)
	≥18 (n=79)	1,838.46 (21,015.65)	55 (69.62%)	2,487.06 (24,383)
**Respiratory management costs**	00-03 (n=6)	- (-)	<5 (-)	- (-)
	04-07 (n=26)	- (-)	<5 (-)	- (-)
	08-12 (n=43)	1,373.09 (13,749.29)	6 (13.95%)	8,859.5 (22,584.55)
	13-17 (n=54)	8,473.41 (33,286.17)	21 (38.89%)	19,038.3 (45,087.04)
	≥18 (n=79)	31,457.51 (151,826.17)	65 (82.28%)	36,727.62 (161,573.28)
**Total costs (€)***	00-03 (n=6)	4,043.96 (16,355.41)	6 (100.00%)	4,043.96 (16,355.41)

04-07 (n=26)	5,888.51 (9,890.67)	26 (100.00%)	5,888.51 (9,890.67)
08-12 (n=43)	22,385.64 (74,866.22)	43 (100.00%)	22,385.64 (74,866.22)
13-17 (n=54)	29,745.16 (96,627.09)	54 (100.00%)	29,745.16 (96,627.09)
≥18 (n=79)	27,516.08 (70,345.26)	79 (100.00%)	27,516.08 (70,345.26)

Abbreviations: ED: emergency department**; HCRU:** healthcare resource use**; PPY** per patient year; **SD**: standard deviation.

Supplementary table 12 The proportion (%) of males remaining in the DMD population formed by the algorithm by inclusion/exclusion criteria

Criteria	Males	Females	% Males
**G71.0, M2Q (first year in which this is fulfilled)**	1402	1115	56%
**At least 1 "intermediate/final diagnosis" G71.0-M1Q***	1159	903	56%
**At least one G71.0 diagnosis in at least two different clinics/hospitals in the first year**	421	241	64%
Age <40	242	75	76%
**Exclusion of patients receiving ventilation ≤ 3 years of age**	240	75	76%
**At least one of the following age-related additional criteria (fulfilled in at least one year of age)**	141	24	85%
**Long-term therapy with glucocorticoids between 4 and 16 years; at least two consecutive quarters of prescription from one of either deflazacort, prednisolone, or prednisone**	35	0	100%
**Wheelchair use <16 years of age**	58	8	88%
**Evidence of walking aid <13 years of age**	17	4	81%
**Cardiomyopathy/ACE inhibitors/Beta-blocker prescription <26 years of age**	72	5	94%
Ventilation ≥10 years old	78	15	84%
**Exclusion of patients with no ventilation at the age of ≥30 years of age**	140	24	85%
Exclusion of females	140	0	100%
**At least 12 months of continuous observation data following the first G71.0-M2Q diagnosis**	134	0	100%

© 2025 – WIG2 GmbH

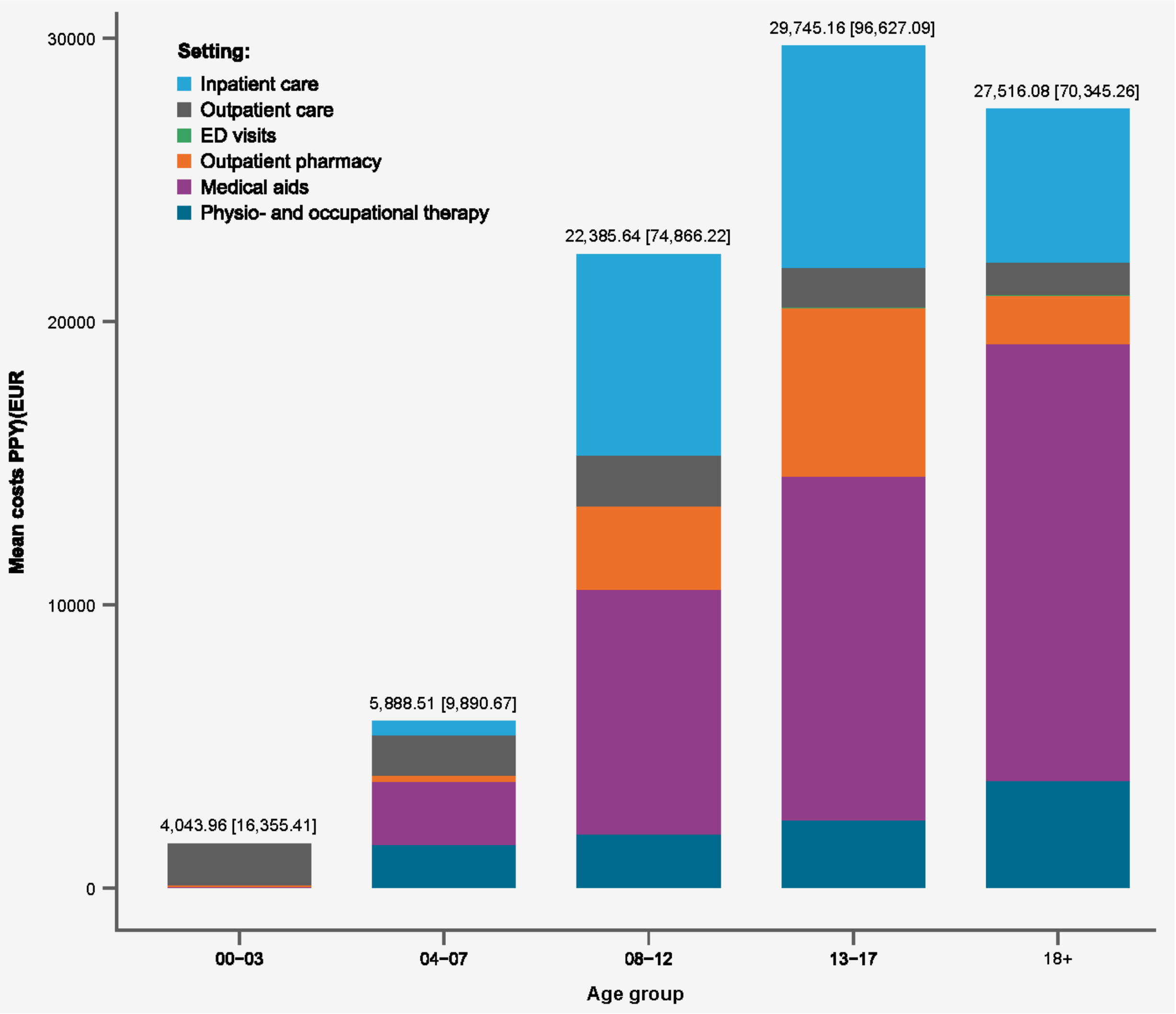

Supplementary figure 1 Mean direct healthcare costs (€) by sector and age group, per patient year (2017-2021)

References

1. Bundesinstitut für Arzneimittel und Medizinprodukte (BfArM). ICD-10-GM. 21/10/2024. https://www.bfarm.de/EN/Code-systems/Classifications/ICD/ICD-10-GM/_node.html. Accessed 21 Oct 2024.
2. Schrader R, Posner N, Dorling P, Senerchia C, Chen Y, Beaverson K, et al. Development and electronic health record validation of an algorithm for identifying patients with Duchenne muscular dystrophy in US administrative claims. J Manag Care Spec Pharm. 2023;29:1033–44. doi:10.18553/jmcp.2023.29.9.1033.
3. Schüssel K, Breitkreuz J, Brückner G, Schröder H. Nutzung von Krankenkassenroutinedaten zur Bestimmung von Krankheitshäufigkeiten im Projekt BURDEN 2020. [Utilizing Routine Health Insurance Data for Calculation of Disease Frequencies in the Project BURDEN 2020]. Gesundheitswesen (Bundesverband der Arzte des

Offentlichen Gesundheitsdienstes (Germany)). 2023;85:S101-S110. doi:10.1055/a-1806-2115.

4. Birnkrant DJ, Bushby K, Bann CM, Alman BA, Apkon SD, Blackwell A, et al. Diagnosis and management of Duchenne muscular dystrophy, part 2: respiratory, cardiac, bone health, and orthopaedic management. Lancet Neurol. 2018;17:347–61. doi:10.1016/S1474-4422(18)30025-5.
5. Broomfield J, Hill M, Guglieri M, Crowther M, Abrams K. Life Expectancy in Duchenne Muscular Dystrophy: Reproduced Individual Patient Data Meta-analysis. Neurology. 2021;97:e2304-e2314. doi:10.1212/WNL.0000000000012910.
6. Schreiber-Katz O, Klug C, Thiele S, Schorling E, Zowe J, Reilich P, et al. Comparative cost of illness analysis and assessment of health care burden of Duchenne and Becker muscular dystrophies in Germany. Orphanet J Rare Dis. 2014;9:210. doi:10.1186/s13023-014-0210-9.
7. Thayer S, Bell C, McDonald C. The Direct Cost of Managing a Rare Disease: Assessing Medical and Pharmacy Costs Associated with Duchenne Muscular Dystrophy in the United States. J Manag Care Spec Pharm. 2017.
8. Orphanet: Das Portal für seltene Krankheiten und Orphan Drugs. Muskeldystrophie Typ Duchenne. https://www.orpha.net/de/disease/detail/98896#menu. Accessed November 2024.
9. Brabec P, Vondrácek P, Klimes D, Baumeister S, Lochmüller H, Pavlík T, Gregor J. Characterization of the DMD/BMD patient population in Czech Republic and Slovakia using an innovative registry approach. Neuromuscul Disord. 2009;19:250–4. doi:10.1016/j.nmd.2009.01.005.
10. Rubin M. Duchenne-Muskeldystrophie und Becker-Muskeldystrophie: Ausgabe für medizinische Fachkreise. 2022. https://www.msdmanuals.com/de-de/profi/p%C3%A4diatrie/angeborene-muskelkrankheiten/duchenne-muskeldystrophie-und-becker-muskeldystrophie. Accessed 01/2022.
11. Duan D, Goemans N, Takeda S, Mercuri E, Aartsma-Rus A. Duchenne muscular dystrophy. Nat Rev Dis Primers. 2021;7:13. doi:10.1038/s41572-021-00248-3.
12. Manzur AY, Muntoni F. Diagnosis and new treatments in muscular dystrophies. J Neurol Neurosurg Psychiatry. 2009;80:706–14. doi:10.1136/jnnp.2008.158329.
13. Janisch M, Boehme K, Thiele S, Bock A, Kirschner J, Schara U, et al. Tasks and interfaces in primary and specialized palliative care for Duchenne muscular dystrophy - A patients' perspective. Neuromuscul Disord. 2020;30:975–85. doi:10.1016/j.nmd.2020.09.031.

14. Soslow JH, Hall M, Burnette WB, Hor K, Chisolm J, Spurney C, et al. Creation of a novel algorithm to identify patients with Becker and Duchenne muscular dystrophy within an administrative database and application of the algorithm to assess cardiovascular morbidity. Cardiol Young. 2019;29:290–6. doi:10.1017/S1047951118002226.
15. LoMauro A, D'Angelo MG, Aliverti A. Assessment and management of respiratory function in patients with Duchenne muscular dystrophy: current and emerging options. Ther Clin Risk Manag. 2015;11:1475–88. doi:10.2147/TCRM.S55889.

© 2025 – WIG2 GmbH
