# Supplementary material for: Epidemiology, disease burden and costs of Duchenne muscular dystrophy in Germany: an observational, retrospective health claims data analysis
Source: Orphanet J Rare Dis. 2025 Aug 13;20:429. doi: 10.1186/s13023-025-03906-x (PMC12351880; doi:10.1186/s13023-025-03906-x)
Supplement: Supplementary file 2 — Additional file2 [file 13023_2025_3906_MOESM2_ESM.docx]

**Supplementary tables (additional material is provided in one pdf)**

Supplementary Table 1 Inclusion and exclusion criteria applied to identify DMD patients with sources from which algorithm was adapted

Supplementary Table 2 Codes used in the algorithm to identify DMD patients in the WIG2 database claims data

Supplementary Table 3 Codes (by category) used to determine disease stage allocation and pre-defined healthcare resource use

Supplementary Table 4 The number of patients (N) included in the DMD study population in total by year, by inclusion/exclusion step

Supplementary Table 5 Longitudinal analysis of the most frequently documented 3-digit ICD-10 GM codes from 2017–2021, by DMD disease stage approximation

Supplementary Table 6 Longitudinal analysis of the most frequently documented 5-digit ATC codes from 2017–2021, by DMD disease stage approximation

Supplementary Table 7 Longitudinal analysis of the most frequently documented 4-digit SHI medical aid codes from 2017–2021, by DMD disease stage approximation

Supplementary Table 8 Longitudinal analysis of the most frequently documented operations/procedures, by 3-digit OPS codes from 2017–2021, by DMD disease stage approximation

Supplementary Table 9 Mean number of HCRU by sector per patient year (PPY), among all patients and among patients with at least 1 HCRU, stratified by disease stage and across the timeframe 2017–2021

Supplementary Table 10 Mean PPY costs by sector (€, with SD) among all patients and among patients with at least 1 HCRU, stratified by disease stage and across the timeframe 2017–2021

Supplementary Table 11 Mean PPY costs by sector (€, with SD) among all patients and among patients with at least 1 HCRU, stratified by age group and across the timeframe 2017–2021

Supplementary Table 12 The proportion (%) of males remaining in the DMD population formed by the algorithm by inclusion/exclusion criteria

Supplementary Fig. 1 Mean direct healthcare costs (€) by sector and age group, per patient year (2017–2021)
